# Integrated proteogenomic analysis revealed the metabolic heterogeneity in noncancerous liver tissues of patients with hepatocellular carcinoma

**DOI:** 10.1186/s13045-021-01195-y

**Published:** 2021-12-11

**Authors:** Haotian Liao, Jinpeng Du, Haichuan Wang, Tian Lan, Jiajie Peng, Zhenru Wu, Kefei Yuan, Yong Zeng

**Affiliations:** 1 Department of Liver Surgery and Liver Transplantation, State Key Laboratory of Biotherapy and Cancer Center, West China Hospital, Sichuan University and Collaborative Innovation Center of Biotherapy, Chengdu, China; 2 School of Computer Science, Northwestern Polytechnical University, Xi’an, China; 3 Key Laboratory of Big Data Storage and Management, Northwestern Polytechnical University, Ministry of Industry and Information Technology, Xi’an, China; 4 Laboratory of Pathology, Department of Pathology, West China Hospital, Sichuan University, Chengdu, China

**Keywords:** Hepatocellular carcinoma, Proteogenomic, Metabolism, Liver microenvironment

## Abstract

Understanding the adjacent liver microenvironment of hepatocellular carcinoma (HCC) with possible metastasis tendency might provide a strategy for risk classification of patients and potential therapies by converting the unique metastasis-inclined microenvironment to a metastasis-averse one. In this study, we performed an integrated proteogenomic analysis to have a comprehensive view on the heterogeneity of hepatic microenvironment contributing to HCC metastasis. Pairing mRNA-protein analysis revealed consistent and discordant mRNA-protein expressions in metabolism regulations and cancer-related pathways, respectively. Proteomic profiling identified three subgroups associated with the recurrence-free survival of patients. These proteomic subgroups demonstrated distinct features in metabolic reprogramming, which was potentially modified by epigenetic alterations. This study raises the point of metabolic heterogeneity in HCC noncancerous tissues and may offer a new perspective on HCC treatment.

**Supplementary Information:**

The online version contains supplementary material available at 10.1186/s13045-021-01195-y.

**To the Editor,**

Hepatocellular carcinoma (HCC) is an aggressive malignancy characterized by high rate of relapse after surgery due to intrahepatic metastasis. Nevertheless, there are still patients who undergo surgical resection having favorable outcomes with no detectable recurrence during follow-up, suggesting the heterogeneity in local microenvironment of noncancerous sites dictating the ability of a tumor to metastasize [1, 2]. On the other hand, HCC is usually present in the background of inflamed fibrotic and/or cirrhotic liver with extensive lymphocyte infiltration caused by chronic hepatitis, indicating that the metastatic propensity of HCC might be influenced by local tissue microenvironment of the host [3]. Indeed, several cellular and acellular components in liver microenvironment have been highlighted in the development or potential regression of HCC hepatic metastasis [4]. Based on the above reasons, understanding the liver microenvironment with possible metastasis tendency might provide a strategy for risk classification of patients and potential therapies of metastatic HCC by converting the unique metastasis-inclined phenotype (MI) to a metastasis-averse (MA) one.

To comprehensively understand the heterogeneity of hepatic microenvironment contributing to HCC metastasis, we performed a proteogenomic analyses integrating epigenomic, transcriptomic and proteomic data. A total of 77 noncancerous liver tissues from 77 HCC patients were selected in current study (Fig. 1a). After pathological inspection and quality controls during library preparation, the availability of methylation, RNA-sequencing (RNA-seq) and proteome data for each HCC candidate is listed in Fig. 1a (lower panel), in which > 96% patients have all of the three omics data.

**Fig. 1:**
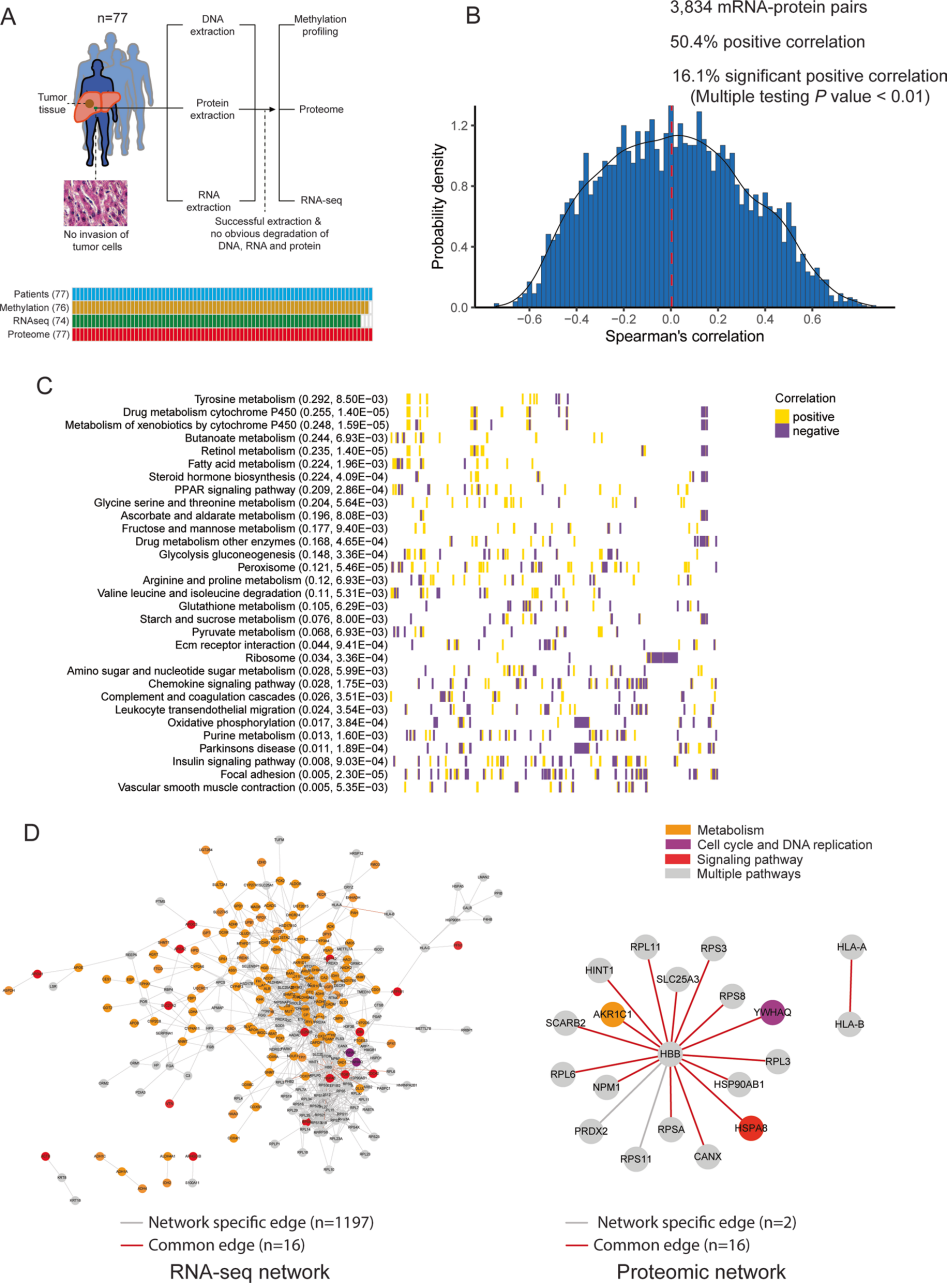
The study design and correlation analyses between mRNA and proteome data. **a** Study workflow. **b** Distribution of correlations for mRNA-protein pairs across 74 samples (the red dashed line indicates the median value of mRNA-protein correlations). **c** GSEA enrichment analysis showed pathways with positive mRNA-protein correlations (Kolmogorov–Smirnov test, Benjamini–Hochberg adjusted *P* < 0.01. The mean correlation was shown in parentheses, which was followed by the adjusted *P* value. Individual proteins in each pathway were represented as bars on the *x* axis, in which golden bars indicate positive correlations, and purple ones indicate negative correlations). **d** Co-expression networks of protein and mRNA data respectively, based on Joint Random Forest (JRF) method

Initially, 6,350 proteins were identified by multiplexed tandem mass tag (TMT) mass spectrometry (MS) with an average of 5,331 in each sample. After removing proteins with missing value in > 50% of the samples, 5,445 proteins with imputed value were selected for the following analyses. Pairing transcriptomic and proteomic data from 74 patients resulted in 3,834 mRNA-protein pairs, which showed an overall correlation close to 0 with only 16.1% (617/3,834) significant positive correlations (multiple-test adjusted *P* < 0.01, Fig. 1b). This fact highly suggested a large amount of post-transcriptional modifications in noncancerous liver tissues. Table S1 (Additional file 3) listed the top 20 genes with the highest mRNA-protein correlations, among which a larger number of metabolism-related genes were identified, such as FN3KRP, GSTT1, AKR1C2, DPYS, SULT2A1 and GSTZ1. Strikingly, genes involved in various metabolic processes had the strongest positive mRNA-protein correlations (Fig. 1c; Additional file 1: Table S2), which was consistent with the findings in HCC tumor tissues [5]. Notably, mRNA-protein pairs with significant discordant expression were involved in various cancer-related pathways (Additional file 1: Fig. S1 and Table S2), which might be owing to the high level of somatic mutation burden in cirrhotic liver [6], potentially leading to the aberrant post-transcriptional regulation of these genes [7]. Despite that co-expression network analysis identified a major functional module consisting of multiple metabolic genes in RNA-seq network, no enrichment module was detected in proteomic network (Fig. 1d). These facts highlighted the necessity of using proteome data to reflect the authentic expression profile of liver microenvironment.

In order to evaluate the heterogeneity and homogeneity among noncancerous samples, we then performed unsupervised clustering based on top 25% most varied proteins among the included samples (Additional file 1: supplementary methods and Fig. S2) and identified three subgroups among the 77 samples (Fig. 2a). While S1 and S2 patients have similar expression patterns, there is a perceptible difference between S3 and each of them (Kruskal–Wallis test). Clinicopathologic parameters such as higher AFP level, tumor thrombus and advanced TNM stages were more prominent in S3 than the other two subgroups. Through manual curation (full list in Table S3, Additional file 3), we found that each subgroup was characterized by various level of metabolism-related proteins. For example, fructose-bisphosphate aldolase C, which is involved in numerous metabolism pathways [8], was ranked in the top 20 proteins in Fig. 2a (Additional file 3: Table S4). To explore this inter-sample heterogeneity, we quantified the activity of a metabolic pathways in each subgroup using a pathway activity score algorithm as previously described [9]. Indeed, significant metabolic heterogeneity was found among these three subgroups, each of which demonstrated a unique profile of metabolic activity (Fig. 2b). By dividing patients into two groups based on the occurrence of early relapse (within 2 years after surgery), we found that the proportion of early relapse samples in S2 subgroups was significantly different from both S1 and S3 subgroups (Fig. 2c, left panel). Moreover, the proteomic subgroups significantly had different recurrence-free survival within 2 years after surgery (Fig. 2c, right panel). Multivariable analysis authenticated the role of proteomic subgroups as independent prognosticators after adjusted for clinicopathologic factors. After stratifying patients according to TNM stage, however, proteomic subgroups showed no correlation with patient prognosis (Fig. 2d, right panel), supporting the effect from tumor tissue on the molecular features of corresponding noncancerous liver tissue. Nevertheless, the proportion of early relapse samples in S2 subgroups was still significantly different from both S1 and S3 subgroups regardless of tumor stages (Fig. 2d, left panel). Meanwhile, the abundance of p62 protein, high expression of which in non-tumor human liver has been proved to be able to predict rapid HCC recurrence after curative treatment [10], was also compared among these three subgroups. The result showed distributed difference of p62 abundance among proteomic subgroups (Fig. 2e), which further validated the findings in our survival analyses. Given that tumor thrombus is one of the most important recurrence-related factors, we compared proteomic profiles between samples with or without tumor thrombus, which revealed 447 differentially expressed proteins (Fig. 2f), among which 31 metabolism-related proteins and p62 protein were identified. Moreover, Gene Set Enrichment Analysis (GSEA) also showed that the differentially expressed proteins were enriched in metabolism-related pathways (Fig. 2g). Based on the above, a crucial dysregulation of metabolism was also observed in noncancerous tissues, which was similar with the situation in tumor tissues [5].

**Fig. 2:**
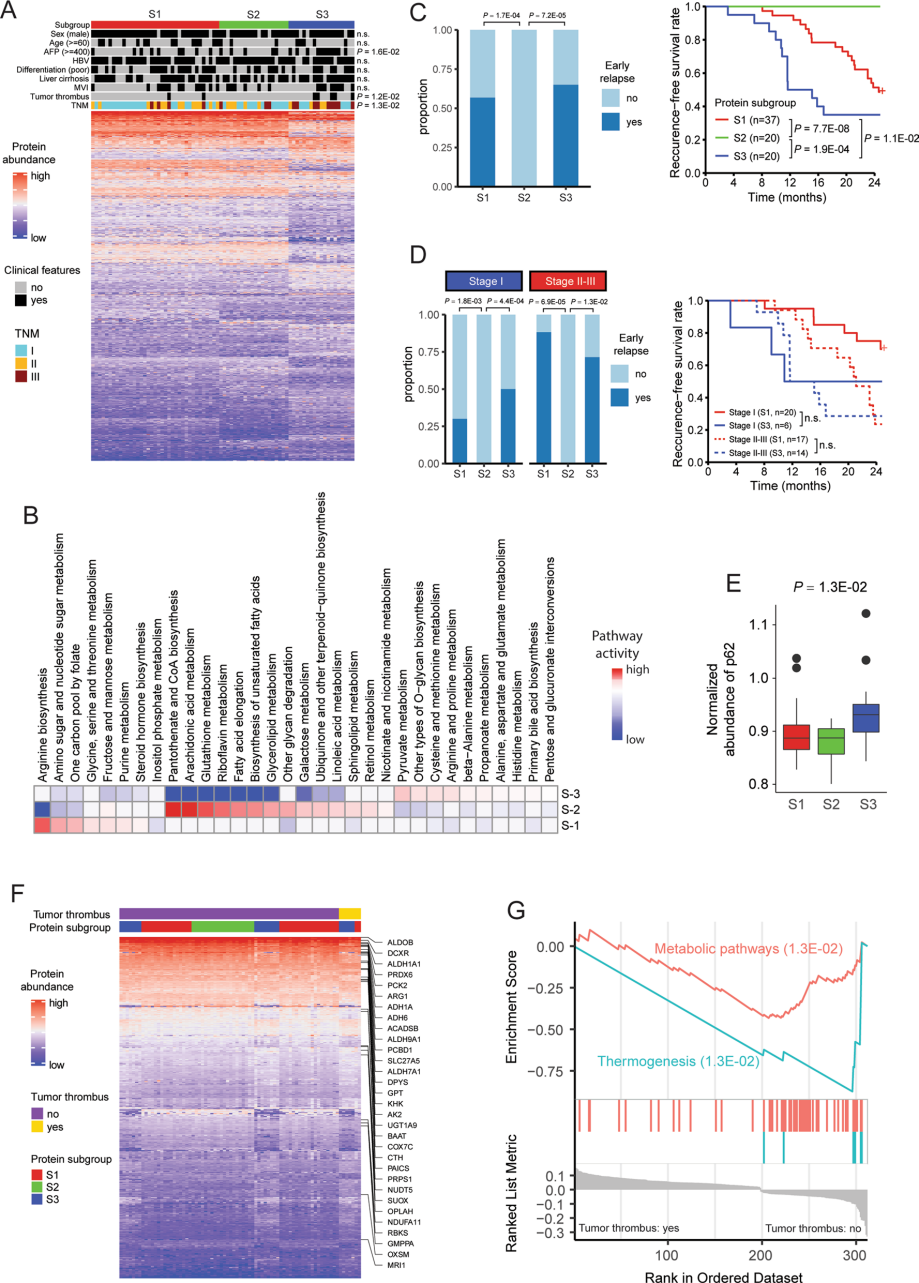
Proteomic stratification of samples included and their clinicopathologic relevance. **a** Heatmap for the differentially expressed proteins among the tree subgroups (*n* = 1,009, Kruskal–Wallis test, Benjamini–Hochberg adjusted *P* < 0.05). **b** Metabolic pathway activities in each proteomic subgroups, with statistically non-significant values (random permutation test *P* > 0.05) shown as blank. **c** Proportion of patients with early relapse in each subgroups (left panel, Fisher’s exact test); Kaplan–Meier curves for recurrence-free survival based on proteomic subgroups (right panel, Log-rank test). **d** Proportion of S1 and S3 subgroups with early relapse at different TNM stages (left panel, Fisher’s exact test); Kaplan–Meier curves for recurrence-free survival of S1 and S3 subgroups at different TNM stages (right panel, Log-rank test). **e** Quantifications of p62 protein in each subgroup (Kruskal–Wallis test). **f**–**g** The heatmap (**f**) and GSEA results (**g**) of significantly differentially expressed proteins (Kruskal–Wallis test, Benjamini–Hochberg adjusted *P* < 0.05) in patients diagnosed with or without tumor thrombus, among which the 31 differentially expressed metabolism-related proteins were labeled on the right side of the heatmap

Although it was not the main purpose of this study, we also conducted a supervised analysis to identify representative prognostic protein markers (Additional file 2: Fig. S3a), which resulted in two proteins, GNG7 (G Protein Subunit Gamma 7) and MCIDAS (Multiciliate Differentiation And DNA Synthesis Associated Cell Cycle Protein), showing differential expression between MI and MA samples (defined by Budhu et al. [3], see Supplementary methods in Additional file 1). Using median as the cutoff (Additional file 1: Supplementary methods), both two proteins demonstrated significant predicting values on the recurrence-free survival, which was further confirmed by multivariable analysis (Additional file 2: Fig. S3b). Regarding the potential function of these two markers, tumors with high GNG7 showed significant upregulation of various pathways relevant to cell signaling and metabolism (Additional file 2: Fig. S3c), while those with high MCIDAS were featured with upregulation of pathways related to tumor progressions, cell cycle, immune response and metabolism (Additional file 2: Fig. S3d).

Intrahepatic metastasis of HCC is accompanied by alterations in the immune status of the tumor-surrounding issue [3], suggesting the potential relationship between HCC recurrence and liver immune microenvironment. To further depict the microenvironment of the noncancerous livers, we enumerated cell subsets in each proteomic subgroup from transcriptomes. While S1 and S2 samples demonstrated similar enrichment for each cell type, perceptible difference was also found in the proportion of cell component between S3 and S1/S2 samples (Additional file 2: Fig. S4a). Since no difference in stroma scores was observed among the three subgroups (Additional file 2: Fig. S4c), the difference in microenvironment score (Additional file 2: Fig. S4d) was mainly contributed by the heterogeneity in immune cell infiltration (Additional file 2: Fig. S4b).

At the same time, we also evaluated the epigenetic regulation of gene expression by using methylation data. A total of 10,9624 differentially methylated regions (DMRs) across all autosomes were detected among the three subgroups (Additional file 2: Fig. S5a), the majority of which are located within intergenic regions (Additional file 2: Fig. S5b). Since hypermethylated DMRs are expected to be associated with decreased gene expression, we analyzed the potential effect of DMRs on mRNA and protein expressions using a Gaussian mixture model, which identified 1,664 proteins significantly attenuated by DMRs regions (Additional file 2: Fig. S5c), corresponding to 43.4% of all the 3,834 genes analyzed. Intriguingly, these attenuated proteins were mainly enriched in various metabolism pathways (Additional file 2: Fig. S5d), which highly suggested that expressions of these metabolic proteins can be regulated by the methylation status of the corresponding genes, which led to the metabolic heterogeneity among the proteomic subgroups. Fig. S6 (Additional file 2) demonstrated the overlaps of results from RNA-seq, methylation profiling and proteome analysis.

In summary, we proved the heterogeneity of HCC noncancerous tissues was caused by dysregulation of metabolism, which was potentially modified by epigenetic alterations and significantly correlated different survival outcomes. These findings revealed the potential of using metabolic inhibitors to prevent early recurrence of HCC after surgery.

## Supplementary Information

**Additional file 1.** Supplementary methods.

**Additional file 2.** Figures S1–S6.

**Additional file 3.** Tables S1–S4.

## Data Availability

All the datasets used and/or analyzed during the current study are available from the corresponding author on reasonable request.

## Abbreviations

HCC: Hepatocellular carcinoma
TMT: Tandem mass tag
MS: Mass spectrometry
RNA-seq: RNA-sequencing
GSEA: Gene Set Enrichment Analysis
DMR: Differentially methylated region
