# Supplementary material for: Integrated proteogenomic analysis revealed the metabolic heterogeneity in noncancerous liver tissues of patients with hepatocellular carcinoma
Source: J Hematol Oncol. 2021 Dec 11;14:205. doi: 10.1186/s13045-021-01195-y (PMC8665512; doi:10.1186/s13045-021-01195-y)
Supplement: Supplementary file 1 — Additional file 1. Supplementary methods. [file 13045_2021_1195_MOESM1_ESM.docx]

**Clinical sample acquisition**

Noncancerous liver tissues from 77 HCC patients who underwent primary curative resection from October 2012 to July 2018 at West China Hospital and received no prior anticancer treatments were enrolled in current study. Samples were collected within 30 min after surgical resection and snap-frozen in liquid nitrogen, then transferred to -80°C within 30 min for later multi-omics analyses. Clinical profiles of patients, including sex, age at diagnosis, hepatitis B infection (positive or negative), pre-operative serum alpha-fetoprotein (AFP) level, were retrieved from the electronic medical database. Besides, characteristics of the paired tumor tissues, including differentiation status, tumor volume, the number of nodules and Ishak fibrosis score of the non-tumor liver tissues, were evaluated by two pathologists specializing in hepatic diseases. Tumor staging classification was performed according to the 8th AJCC TNM Staging for Liver and Intrahepatic Bile Duct Malignancies. The study protocols were approved by the ethical community of West China Hospital and all patients provided written informed consent. Metastasis-inclined (MI) refers to hepatic tissues from patients with one of the following conditions: 1) venous metastases found in the portal vein, inferior vena cava, or common bile duct; 2) multifocal lesions at diagnosis; 3) intrahepatic metastasis within 2 years after surgery(1, 2); 4) distant metastases that were confirmed at follow-up. Metastasis-averse (MA) refers to hepatic tissues from patients carrying a single HCC lesion with no detectable metastases at the time of diagnosis and at follow-up within 2 years after surgery.

**DNA methylation data**

DNA from noncancerous tissues was extracted using the DNeasy Blood & Tissue Kit (Qiagen, Venlo, Limburg, The Netherlands) according to the manufacturer’s instruction. The quality of isolated genomic DNA was quantified and verified by Qubit® DNA Assay Kit in Qubit® 2.0 Flurometer (Invitrogen, Carlsbad, CA, USA) and agarose gel electrophoresis. DNA methylation was characterized using Infinium HumanMethylationEPIC BeadChip Kit (Illumina), which interrogates over 850,000 CpG sites. Raw data in .idat format were imported and processed in R (version 3.5.2) using minfi (version 1.30.0) and ChAMP (version 2.14.0) packages. Probes with the following features were excluded from further analysis: 1) median detection *P* value ≥ 0.01; 2) number of beads < 3 in over 5% of the samples; 3) mapping to sex chromosomes; 4) non-CG; 5) multi-hit 6) within 5 bp of a known single-nucleotide polymorphism (SNP). The DNA methylation intensity at a specific CpG site was evaluated as β=M/(M+U+100)^−1^, in which M and U denote methylated and unmethylated fluorescent signal intensities, respectively. Data normalization was performed using BMIQ (Beta MIxture Quantile dilation) method. Differentially methylated regions (DMRs) between the three proteomic subgroups were called using R package DMRcate(3) (version 1.18.0) with ANOVA mode. DMRs were defined as those with Stouffer-transformed limma-derived BH *P* value < 0.05 adjusted for covariates including sex, age, AFP level, HBV infection, tumor differentiation, TNM, tumor size, tumor number, and the existence of liver cirrhosis, MVI and tumor thrombus. The cut-off number of CpG sites in each DMR is 2. The methylation status of each DMR was determined as the averaged β values of probes in this DMR.

**RNA expression**

Total RNA was extracted and purified from noncancerous liver tissues using the Trizol reagent (Invitrogen). RNA integrity was confirmed on the Agilent 2100 Bioanalyzer (Agilent Technologies). After total RNA extraction, mRNA was isolated by Oligo Magnetic Beads and cut into small fragments for cDNA synthesis. The NEBNext® Ultra™ RNA Library Prep Kit for the Illumina system (New England Biolabs, Ipswich, MA, USA) was used for Library generation according to the manufacturer’s instructions. The cDNA synthesis, end-repair, A-base addition, and ligation of the Illumina index adapters were performed according to Illumina’s TruSeq RNA protocol (Illumina). Library quality was measured on an Agilent 2100 Bioanalyzer for product size and concentration. Paired-end libraries (2 × 150-nucleotide read length) were sequenced by Illumina HiSeq X Ten system, with a sequence coverage of 40 million paired reads. Raw data were processed through quality control step to obtain clean data with high quality. For the quantification of gene expressions, paired-end clean reads were aligned to the reference genome (hg19) using Hisat2 v2.0.5. After reads mapping, HTSeq was used to count the reads numbers mapped to each gene. Then FPKM value was calculated based on gene length and reads count mapped to this gene. Finally, the log_2_ FPKM values were used for the following analysis.

**Quantitative proteomics by multiplexed tandem mass tag (TMT) MS**

The noncancerous liver tissues were individually ground in liquid nitrogen and lysed in a buffer containing 100 mM NH4HCO3(pH 8), 8M Urea and 0.2% SDS, which was followed by 5 min of ultrasonication on ice. The lysate was centrifuged at 12,000 g for 15 min at 4℃ and the supernatant was transferred to a clean tube. Extracts from each sample were reduced with 10 mM DTT for 1 h at 56℃, and subsequently alkylated with sufficient iodoacetamide for 1 h at room temperature in the dark. Then samples were completely mixed with 4 times volume of precooled acetone by vortexing and incubated at -20℃ for at least 2 h. Samples were then centrifuged and the precipitation was collected. After washing twice with cold acetone, the pellet was dissolved by dissolution buffer, which containing 0.1 M triethylammonium bicarbonate (TEAB, pH 8.5) and 6 M urea. After quality test of sample protein solution, 120 μg of each protein sample was taken and the volume was made up to 100 μL with dissolution buffer and 1.5 μg trypsin (Promega, Madison, WI, USA), in which 500 μL of 100 mM TEAB buffer were added. Each protein sample was digested at 37 °C for 4h. Afterwards, 1.5 μg trypsin and CaCl2 were added, and the sample was then digested overnight. After protein digestion, formic acid was mixed with digested sample (pH < 3) and centrifuged at 12,000 g for 5 min at room temperature. The supernatant was slowly loaded to the C18 desalting column, washed with washing buffer (0.1% formic acid, 3% acetonitrile) 3 times, then eluted by some elution buffer (0.1% formic acid, 70% acetonitrile). The eluent of each sample was combined and lyophilized.

In order to construct data-dependent acquisition (DDA) spectrum library, mobile phase A (2% acetonitrile, adjusted pH to 10.0 using ammonium hydroxide) and B (98% acetonitrile, adjusted pH to 10.0 using ammonium hydroxide) were used to develop a gradient elution. The lyophilized powder was dissolved in solution A and centrifuged at 12,000 g for 10 min at room temperature. The sample was fractionated using a C18 column (Waters BEH C18 4.6×250 mm, 5 μm) on a Rigol L3000 HPLC system, the column oven was set as 50°C. The eluates were monitored at UV 214 nm, collected for a tube per minute and combined into 4 fractions finally. All fractions were dried under vacuum, and then, reconstituted in 0.1% (v/v) formic acid (FA) in water. For transition library construction, shotgun proteomics analyses were performed using an EASY-nLC^TM^ 1200 UHPLC system (Thermo Fisher) coupled with an Q Exactive HF-X mass spectrometer (Thermo Fisher) operating in the data-dependent acquisition (DDA) mode. 1 μg sample containing standard peptides was injected into a home-made C18 Nano-Trap column (2 cm×75 μm, 3 μm). Peptides were separated in a home-made analytical column (15 cm×150 μm, 1.9 μm) using a linear gradient elution. The separated peptides were analyzed by Q Exactive HF-X mass spectrometer (Thermo Fisher), with ion source of Nanospray Flex^TM^ (ESI), spray voltage of 2.5 kV and ion transport capillary temperature of 320°C. Full scan range from 350 m/z to 1500 m/z with resolution of 120,000 @ 200 m/z, an automatic gain control (AGC) target value was 3×10^6^ and a maximum ion injection time was 80 ms. The top 40 precursors of the highest abundant in the full scan were selected and fragmented by higher energy collisional dissociation (HCD) and analyzed in MS/MS, where resolution was 15,000 @ 200 m/z, the automatic gain control (AGC) target value was 5×10^4^, the maximum ion injection time was 45 ms, a normalized collision energy of 27%, an intensity threshold of 1.1×10^4^, and the dynamic exclusion parameter of 20s. The raw data of MS detection was used to construct DDA spectrum library.

For data-independent acquisition (DIA) based liquid Chromatography with tandem mass spectrometry (LC-MS/MS), mobile phases A (0.1% FA in H2O) and B (0.1% FA in 80% ACN) were used to develop a gradient elution. The lyophilized powder of each sample was dissolved in 12 μL of A solution, centrifuged at 15,000 rpm for 20 min at 4 ℃, and each supernatant was added to 0.4 μL of the standard peptide, and then 1 μg of the sample was injected into the EASY-nLC^TM^ 1200 UHPLC system (Thermo Fisher) coupled with an Orbitrap Q Exactive HF-X mass spectrometer (Thermo Fisher) operating in the DIA mode with spray voltage of 2.5 kV, Nanospray Flex™（ESI）and capillary temperature of 320°C. For DIA acquisition, the m/z range covered from 350 to 1,500 with 36 scan windows. MS1 resolution was set to 60,000 @ 200 m/z, full scan AGC target value was 5×10^5^, the maximum ion injection time was 20 ms. Peptides were fragmented by HCD in MS2, in which resolution was set to 30,000 @ 200 m/z, AGC target value was 1×10^6^, a normalized collision energy of 27%.

For protein Identification, both DDA and DIA data were analyzed using Proteome Discoverer 2.2 (PD 2.2, Thermo) platform, Biognosys Spectronaut version 9.0, and R statistical framework. DDA MS raw files were analyzed by PD software (version 2.2) and peak lists were searched against protein database. Cysteine carbamidomethylation was set as a fixed modification and N-terminal acetylation and methionine oxidation as variable modifications. The false discovery rate was set to 1% for both proteins and peptides, and was determined by searching a protein reverse database. The enzyme specificity was set to trypsin (enabling cleavage before proline), and a maximum of two missed cleavages were allowed in the database search. Peptide identification was performed with an allowed initial precursor mass deviation up to 10 ppm and an allowed fragment mass deviation of 10 ppm.

MS1-based label free quantification (LFQ) was done using maxLFQ algorithm to quantify of the proteome data (4). MS2-based-label free quantification was carried out by analyzing DIA raw data using Biognosys Spectronaut (version 9.0) software. Data analysis was carried out as previously described(5) with the following modifications: data extraction and extraction window were set to “dynamic” with correction factor 1, identification was set to “normal distribution p-value estimator” with q-value cutoff of 0.01, and the profiling strategy was set to “iRT profiling” with q value cutoff of 0.01. Ultimately, protein inference was set to “from search engine”, protein quantity was set to “Average precursor quantity” and smallest quantitative unit was set to “Precursor ion” (summed fragment ions). K-nearest neighbor (k-NN) imputation was performed using impute package in R to impute the missing values. Proteins with more than 50% missing data were excluded to ensure sufficient data in each sample for imputation.

**mRNA-protein correlation**

The correlation between mRNA expression and protein abundance for each gene-protein pair across all the samples with both proteome and RNA-seq data was measured by applying Spearman correlation coefficient, in which the respective *P* value for each correlation coefficient was computed and adjusted by Benjamini-Hochberg (BH) FDR correction. An adjusted *P* value cut-off of 0.05 was used to determine significance of the correlation pairs. The interaction patterns among genes and proteins were analyzed by constructing co-expression network based on the global proteomic and RNA-seq data across samples. The top 15% expressed mRNAs and proteins within the largest interquartile range were chosen and generated into two 307×74 data matrices according to the matched gene-protein pairs, in which one was for gene expression and the other was for protein expression. We utilized Joint Random Forest (JRF) method (R package JRF, version 0.1-4) to join the two co-expression networks, which enables an accurate estimation based on information shared in both RNA-seq and proteomic and data. The parameters set for JRF were as following: 1, the total number of trees: 1,000; 2, the number of variables sampled at each node: sqrt (No. of genes -1). FDR of importance scores was calculated with 400 permutations, in which < 0.05 was considered as statistically significant. Genes in both existing mRNA and protein co-expression network edges were mapped to pathways and imported into Cytoscape (https://cytoscape.org/) to create co-expression networks.

**Consensus clustering for proteomic data**

The top 25% most varied proteins within samples with available proteome data were chosen for subgrouping. K-means consensus clustering was then performed on the selected proteins to generate subgroups by using R package ConsensusClusterPlus (version 1.54.0), and the parameters for clustering was set as following: 1, number of repetitions: 1,000 bootstraps; 2, pItem: 0.8 (resampling 80% of any sample); 3, pFeature: 0.8 (resampling 80% of any protein); 4, lower limit of cluster number: 2; 5, upper limit of cluster number: 6. Consideration on the selection of cluster number was based on three factors, including the average pairwise consensus matrix within consensus clusters, the delta plot depicting the relative change in the area under the cumulative distribution function (CDF) curve, and the average silhouette distance for consensus clusters.

**Calculation of metabolic pathway activity**

The activity of each metabolic pathway was calculated as previously described(6). Briefly, the computing process consists of three steps: 1, calculate the mean expression of each metabolic gene (expressed protein) across cells of each proteomic subgroup; 2, quantify the relative expression level of each metabolic gene in each proteomic subgroup comparing to the average expression level of this gene in all proteomic subgroups, in which a quantification value >1 means that expression level of this gene is higher in certain proteomic subgroup compared to its average expression level over all proteomic subgroups; 3, calculation of weighted average of the quantification value of this gene (obtained from step 2) over all genes included in the corresponding pathway, represents the activity of the corresponding pathway. Outliers in each pathway (defined as genes with relative expression levels greater than three times 75th percentile or below 1/3 times 25th percentile) were excluded to avoid the affection on pathway activity scores owing to genes with low expression level or high drop-out rates. Statistical significance of higher or lower pathway activity in a specific subgroup was then evaluated by a random permutation test to assess if activity of certain pathway is significantly higher or lower in this subgroup than average.

**Identification of prognostic proteins**

We chose logistic regression to identify proteins correlated with either MA of MI phenotype. Patients were divided into two groups and the median value of each protein were used as cutoff to define high and low expression of the corresponding protein. The filter criteria for survival analysis were as follows: MI vs. MA with t test adjusted *P* value < 0.05 and fold change≥ 2, variance in all the samples > 0.5, Log-rank *P* value < 0.05 and logistic *P* value < 0.05. Protein abundance across 77 noncancerous samples were subjected to single-sample gene set enrichment (ssGSEA) to achieve enrichment scores over MSigDB (v.6.2) c2 (canonical gene sets, https://software.broadinstitute.org/gsea/msigdb/index.jsp) pathway database with at least 10 overlapping genes by using R package GSVA(7). The significance of the pathway enrichment scores (PES) over high- and low-expression samples was estimated by linear model and moderated with the F-statistic using the R package limma(8). The resulting significant PES among different subsets of patients (low expression vs. high expression) were corrected by the Benjamini-Hochberg method, in which an adjusted *P* value of 0.05 was considered as statistically significant.

**Cell type quantification**

Cell type quantification in each proteomic subgroups was determined by using a gene signature-based method(9). A microenvironment score was generated as the sum of all immune and stromal cell types.

**Gene Set Enrichment Analysis (GSEA)**

Gene Set Enrichment Analysis (GSEA) for the differentially expressed proteins was performed by the GSEA software (http://software.broadinstitute.org/gsea/index.jsp). Gene sets used in this work were c2.cp.kegg.v7.4.symbols.gmt downloaded from the Molecular Signatures Database (MSigDB, <http://software.broadinstitute.org/gsea/msigdb/index.jsp>).

**REFERENCES**

1. Portolani N, Coniglio A, Ghidoni S, Giovanelli M, Benetti A, Tiberio GAM, et al. Early and late recurrence after liver resection for hepatocellular carcinoma: prognostic and therapeutic implications. Ann Surg. 2006;243(2):229-35.

2. Wu JC, Huang YH, Chau GY, Su CW, Lai CR, Lee PC, et al. Risk factors for early and late recurrence in hepatitis B-related hepatocellular carcinoma. Journal of hepatology. 2009;51(5):890-7.

3. Peters TJ, Buckley MJ, Statham AL, Pidsley R, Samaras K, V Lord R, et al. De novo identification of differentially methylated regions in the human genome. Epigenetics & Chromatin. 2015;8(1):6.

4. Cox J, Hein MY, Luber CA, Paron I, Nagaraj N, Mann M. Accurate proteome-wide label-free quantification by delayed normalization and maximal peptide ratio extraction, termed MaxLFQ. Molecular & cellular proteomics : MCP. 2014;13(9):2513-26.

5. Bruderer R, Bernhardt OM, Gandhi T, Miladinović SM, Cheng LY, Messner S, et al. Extending the limits of quantitative proteome profiling with data-independent acquisition and application to acetaminophen-treated three-dimensional liver microtissues. Molecular & cellular proteomics : MCP. 2015;14(5):1400-10.

6. Xiao Z, Dai Z, Locasale JW. Metabolic landscape of the tumor microenvironment at single cell resolution. Nature communications. 2019;10(1):3763.

7. Hänzelmann S, Castelo R, Guinney J. GSVA: gene set variation analysis for microarray and RNA-Seq data. BMC Bioinformatics. 2013;14(1):7.

8. Ritchie ME, Phipson B, Wu D, Hu Y, Law CW, Shi W, et al. limma powers differential expression analyses for RNA-sequencing and microarray studies. Nucleic acids research. 2015;43(7):e47.

9. Aran D, Hu Z, Butte AJ. xCell: digitally portraying the tissue cellular heterogeneity landscape. Genome Biology. 2017;18(1):220.
