# Supplementary material for: Integrated proteogenomic analysis revealed the metabolic heterogeneity in noncancerous liver tissues of patients with hepatocellular carcinoma
Source: J Hematol Oncol. 2021 Dec 11;14:205. doi: 10.1186/s13045-021-01195-y (PMC8665512; doi:10.1186/s13045-021-01195-y)
Supplement: Supplementary file 2 — Additional file 2. Figures S1–S6. [file 13045_2021_1195_MOESM2_ESM.docx]

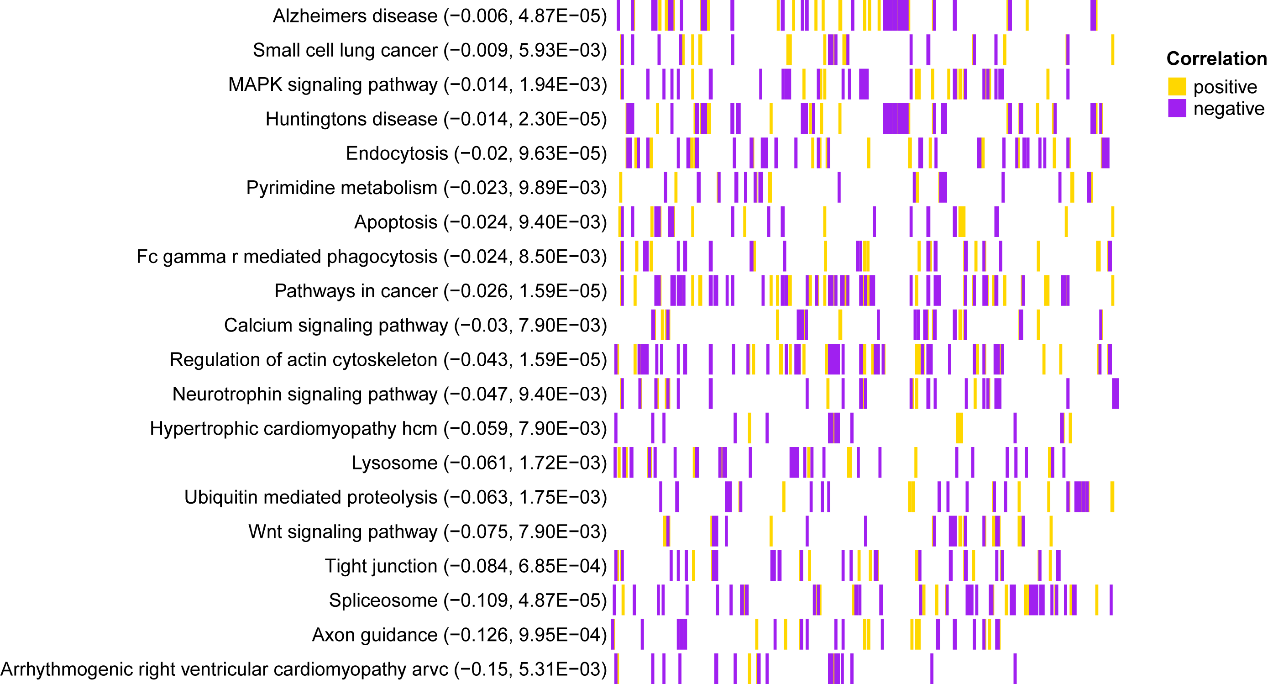


**Fig. S1.** GSEA enrichment analysis showed pathways with negative mRNA-proteins correlations (Kolmogorov-Smirnov test, Benjamini-Hochberg adjusted *P* < 0.01). The mean correlation was shown in parentheses, which was followed by the adjusted *P* value. Individual proteins in each pathway were represented as bars on the x axis, in which golden bars indicate positive correlations, and purple ones indicate negative correlations.


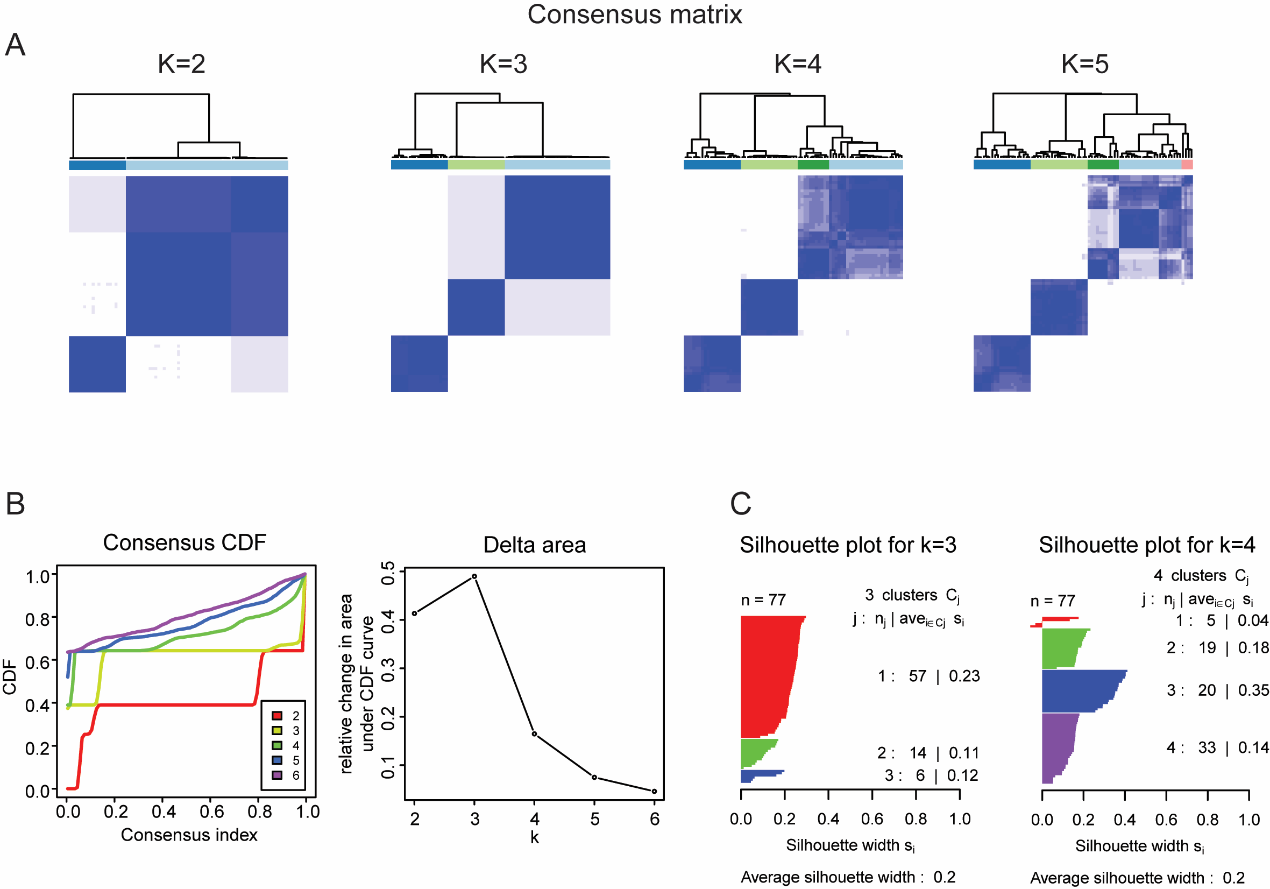


**Fig. S2.** Subgrouping based on proteomic data by K-means consensus clustering upon their abundance, in which k was tested from 2 to 5 and consensus clustering was based on 1,000 resampled datasets. **a** Consensus matrices. **b** Consensus cumulative distribution function (CDF) plot and delta area (change in CDF area) plot. **c** Silhouette plots (k = 3&4). 3-cluster was selected as the best solution for the consensus matrix since k = 3 or k = 4 deemed to be a cleanest separation among clusters, while k = 3 demonstrated a cleaner separation than k = 4 did. Despite that consensus CDF and delta plot exhibited that there was little increase in area for k = 4 compared to k = 3, k = 3 did not have significant negative values while k = 4 did. Based on the evidence above, the proteomic data were clustered into 3 groups. Thus, the decision was finally attributed to (1) a cleaner separation when k = 3 and (2) no silhouette widths with significant negative values observed for 3 clusters.

**
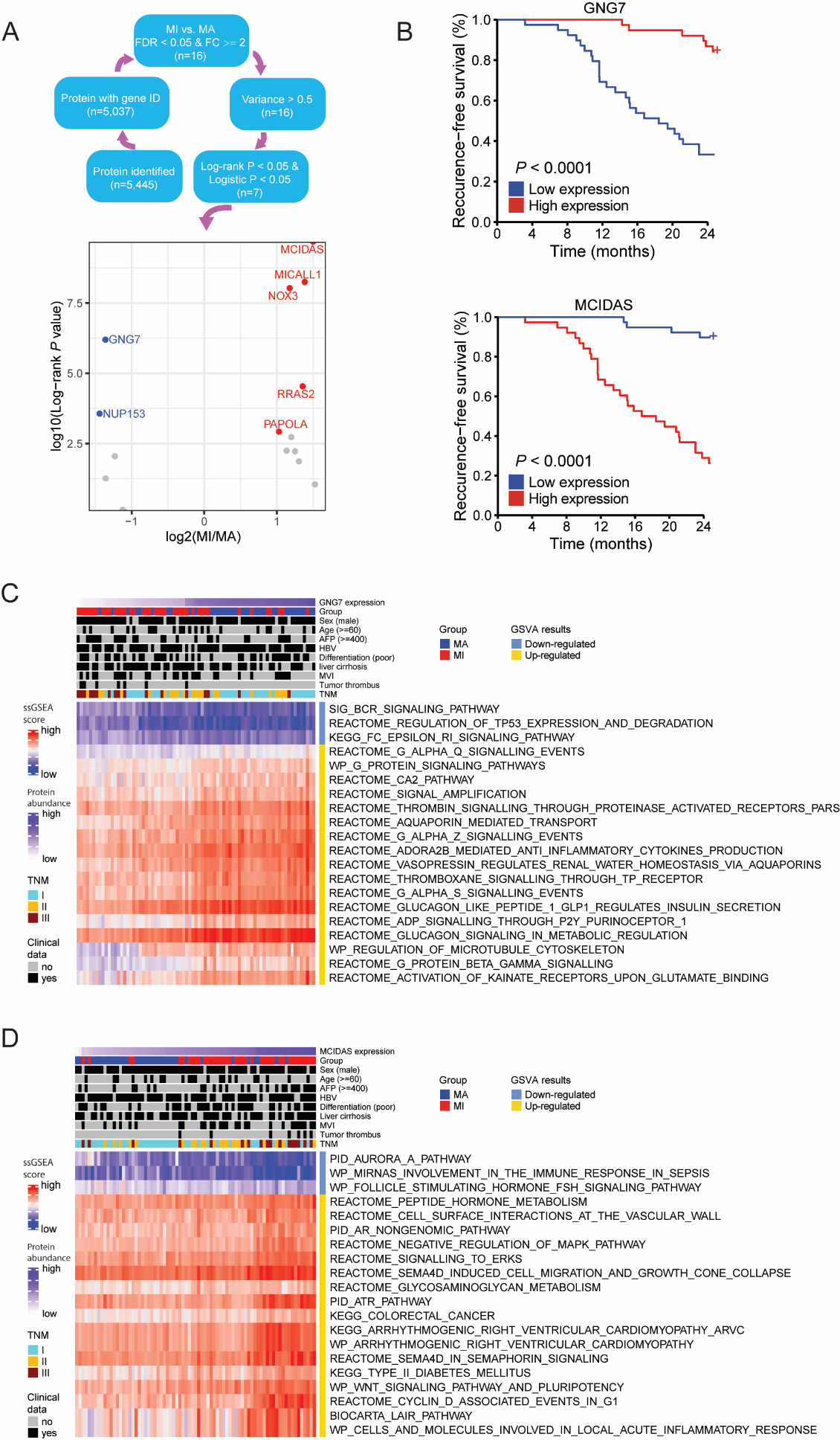
**

**Fig. S3.** Identification of prognostic biomarkers from proteome data. **a** Workflow for prognostic protein selection, with dot representing the 7 candidate proteins (marked in red and blue). FC, fold change; FDR, false discovery rate; MI, metastasis-inclined; MA, metastasis-averse. **b** Kaplan-Meier curves for recurrence-free survival based on proteomic abundance of GNG7 and MCIDAS (Log-rank test). **c-d** ssGSEA analysis shows the associations of GNG7 (c) and MCIDAS (d) expression with proteome profile (only the top 20 pathways were listed for each protein).


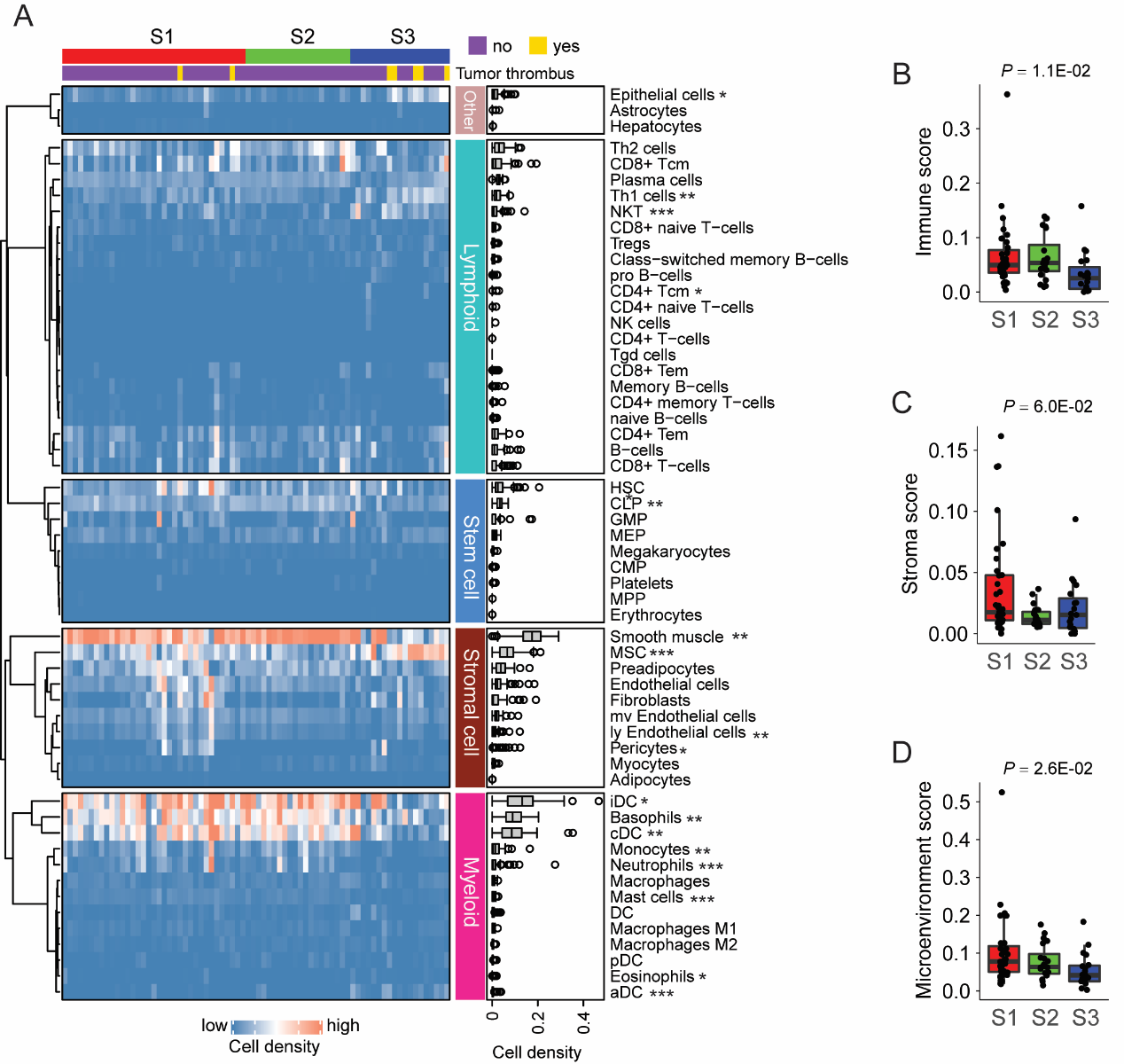


**Fig. S4.** Analysis on the immue microenvironment of each subgroup. **a** Heatmap for the comparison of cell type quatifications among the three proteomic subgroups (Kruskal-Wallis test with Benjamini-Hochberg adjusted *P*); *, *P* < 0.05; **, *P* < 0.01; ***, *P* < 0.001. **b-d** Distribution of immune scores, stroma scores and microenvironment scores in each subgroups (Kruskal-Wallis test).


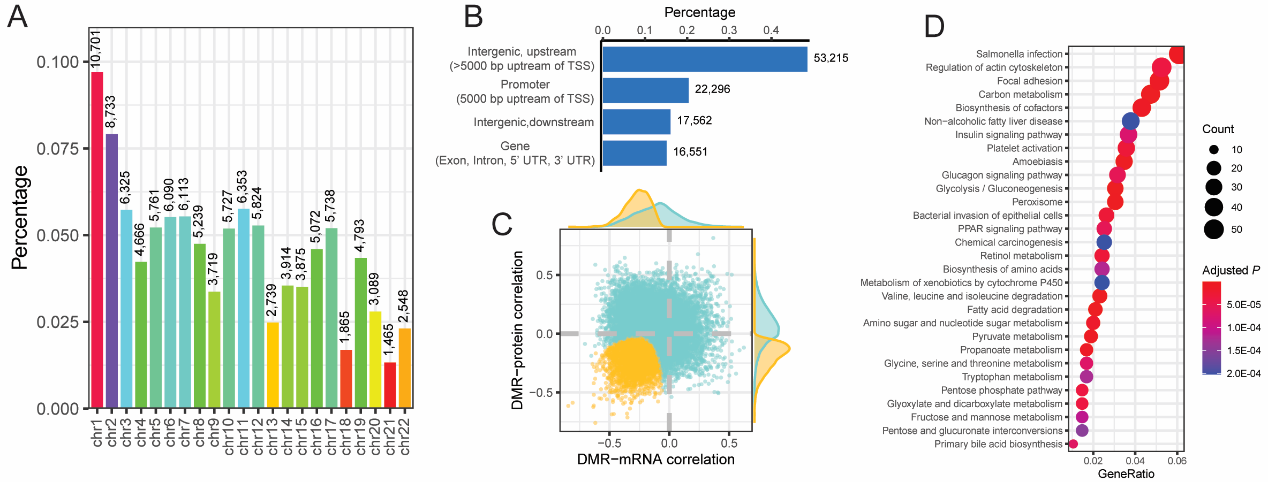


**Fig. S5** Indentification of DMRs among the three subgroups. **a** Numbers of identified DMRs in each chromosome. **b** Genomic distribution of DMRs by relationship to gene. **c** Scatterplot of DMR correlation to mRNA and protein (Spearman’s correlation). Each dot represents a transcript/protein. Significant attenuated proteins are represented in gold using a Gaussian mixture model with two mixture components. **d** Kyoto Encyclopedia of Genes and Genomes (KEGG) pathway enrichment analyses on the significant attenuated proteins identified in **c**. The size of each dot represents the count of enriched genes in the corresponding pathway, and x axis represents the ratio of enriched gene number to that of all genes in the pathway.


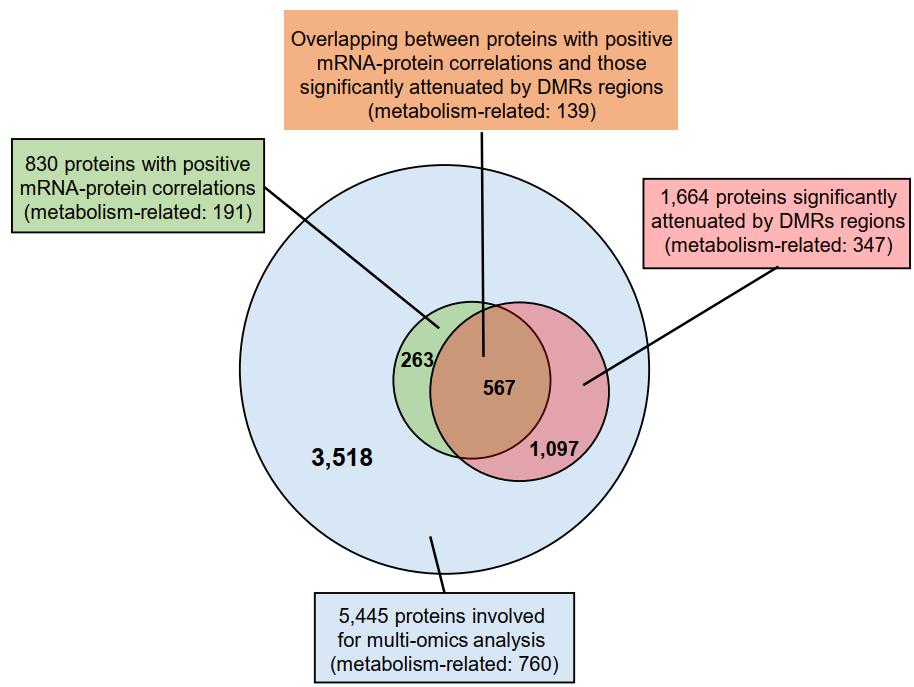


**Fig. S6** Venn Diagram shows the overlaps of results from RNA-seq, methylation profiling and proteome analysis.
