# Supplementary material for: Integrated proteogenomic analysis revealed the metabolic heterogeneity in noncancerous liver tissues of patients with hepatocellular carcinoma
Source: J Hematol Oncol. 2021 Dec 11;14:205. doi: 10.1186/s13045-021-01195-y (PMC8665512; doi:10.1186/s13045-021-01195-y)
Supplement: Supplementary file 3 — Additional file 3. Tables S1–S4. [file 13045_2021_1195_MOESM3_ESM.docx]

| **Table S1. Top 20 genes with highest mRNA-protein correlations** | | |
| --- | --- | --- |
| **Gene symbol** | ***Rho*^1^** | **BH adjusted *P*^2^** |
| FN3KRP | 0.857 | 0 |
| CORO1A | 0.813 | 0 |
| EFHD1 | 0.792 | 0 |
| ASB9 | 0.792 | 0 |
| SOD1 | 0.786 | 0 |
| GSTT1 | 0.782 | 1.26E-14 |
| TMEM120A | 0.776 | 0 |
| TSPAN8 | 0.772 | 0 |
| AKR1C2 | 0.766 | 0 |
| DPYS | 0.761 | 0 |
| SULT2A1 | 0.759 | 0 |
| GSTZ1 | 0.755 | 0 |
| SRD5A2 | 0.754 | 0 |
| GSTM1 | 0.748 | 1.23E-12 |
| HPD | 0.745 | 0 |
| HINT3 | 0.742 | 0 |
| VSNL1 | 0.727 | 0 |
| COX7C | 0.723 | 0 |
| ADH6 | 0.719 | 0 |
| LGALS4 | 0.714 | 0 |

^1^Spearman's rank correlation coefficient of the mRNA-protein pair for the corresponding gene.

^2^Benjamini-Hochberg adjusted *P* value from Spearman's rank correlation test.

| **Table S2. mRNA-protein correlations in all the GSEA enrichment KEGG pathways** | | | |
| --- | --- | --- | --- |
| **Pathway** | **Mean *Rho*^1^** | **KS *P*^2^** | **BH adjusted *P*^3^** |
| KEGG_GLYCOLYSIS_GLUCONEOGENESIS | 0.148 | 2.67E-05 | 3.36E-04 |
| KEGG_RETINOL_METABOLISM | 0.235 | 1.52E-07 | 1.40E-05 |
| KEGG_DRUG_METABOLISM_CYTOCHROME_P450 | 0.255 | 1.17E-07 | 1.40E-05 |
| KEGG_METABOLISM_OF_XENOBIOTICS_BY_CYTOCHROME_P450 | 0.248 | 4.32E-07 | 1.59E-05 |
| KEGG_REGULATION_OF_ACTIN_CYTOSKELETON | -0.043 | 3.71E-07 | 1.59E-05 |
| KEGG_PATHWAYS_IN_CANCER | -0.026 | 3.47E-07 | 1.59E-05 |
| KEGG_FOCAL_ADHESION | 0.005 | 8.01E-07 | 2.30E-05 |
| KEGG_HUNTINGTONS_DISEASE | -0.014 | 8.77E-07 | 2.30E-05 |
| KEGG_SPLICEOSOME | -0.109 | 2.38E-06 | 4.87E-05 |
| KEGG_ALZHEIMERS_DISEASE | -0.006 | 2.23E-06 | 4.87E-05 |
| KEGG_PEROXISOME | 0.121 | 2.97E-06 | 5.46E-05 |
| KEGG_ENDOCYTOSIS | -0.020 | 5.76E-06 | 9.63E-05 |
| KEGG_PARKINSONS_DISEASE | 0.011 | 1.23E-05 | 1.89E-04 |
| KEGG_PPAR_SIGNALING_PATHWAY | 0.209 | 2.02E-05 | 2.86E-04 |
| KEGG_RIBOSOME | 0.034 | 2.74E-05 | 3.36E-04 |
| KEGG_OXIDATIVE_PHOSPHORYLATION | 0.017 | 3.34E-05 | 3.84E-04 |
| KEGG_STEROID_HORMONE_BIOSYNTHESIS | 0.224 | 3.77E-05 | 4.09E-04 |
| KEGG_DRUG_METABOLISM_OTHER_ENZYMES | 0.168 | 4.55E-05 | 4.65E-04 |
| KEGG_TIGHT_JUNCTION | -0.084 | 7.07E-05 | 6.85E-04 |
| KEGG_INSULIN_SIGNALING_PATHWAY | 0.008 | 9.81E-05 | 9.03E-04 |
| KEGG_ECM_RECEPTOR_INTERACTION | 0.044 | 1.07E-04 | 9.41E-04 |
| KEGG_AXON_GUIDANCE | -0.126 | 1.19E-04 | 9.95E-04 |
| KEGG_PURINE_METABOLISM | 0.013 | 2.00E-04 | 1.60E-03 |
| KEGG_LYSOSOME | -0.061 | 2.25E-04 | 1.72E-03 |
| KEGG_CHEMOKINE_SIGNALING_PATHWAY | 0.028 | 2.38E-04 | 1.75E-03 |
| KEGG_UBIQUITIN_MEDIATED_PROTEOLYSIS | -0.063 | 2.48E-04 | 1.75E-03 |
| KEGG_MAPK_SIGNALING_PATHWAY | -0.014 | 2.85E-04 | 1.94E-03 |
| KEGG_FATTY_ACID_METABOLISM | 0.224 | 2.99E-04 | 1.96E-03 |
| KEGG_COMPLEMENT_AND_COAGULATION_CASCADES | 0.026 | 5.53E-04 | 3.51E-03 |
| KEGG_LEUKOCYTE_TRANSENDOTHELIAL_MIGRATION | 0.024 | 5.77E-04 | 3.54E-03 |
| KEGG_VALINE_LEUCINE_AND_ISOLEUCINE_DEGRADATION | 0.110 | 9.09E-04 | 5.31E-03 |
| KEGG_ARRHYTHMOGENIC_RIGHT_VENTRICULAR_CARDIOMYOPATHY_ARVC | -0.150 | 9.24E-04 | 5.31E-03 |
| KEGG_VASCULAR_SMOOTH_MUSCLE_CONTRACTION | 0.005 | 9.59E-04 | 5.35E-03 |
| KEGG_GLYCINE_SERINE_AND_THREONINE_METABOLISM | 0.204 | 1.04E-03 | 5.64E-03 |
| KEGG_SMALL_CELL_LUNG_CANCER | -0.009 | 1.13E-03 | 5.93E-03 |
| KEGG_AMINO_SUGAR_AND_NUCLEOTIDE_SUGAR_METABOLISM | 0.028 | 1.17E-03 | 5.99E-03 |
| KEGG_GLUTATHIONE_METABOLISM | 0.105 | 1.27E-03 | 6.29E-03 |
| KEGG_ARGININE_AND_PROLINE_METABOLISM | 0.120 | 1.47E-03 | 6.93E-03 |
| KEGG_PYRUVATE_METABOLISM | 0.068 | 1.50E-03 | 6.93E-03 |
| KEGG_BUTANOATE_METABOLISM | 0.244 | 1.51E-03 | 6.93E-03 |
| KEGG_CALCIUM_SIGNALING_PATHWAY | -0.030 | 1.85E-03 | 7.90E-03 |
| KEGG_WNT_SIGNALING_PATHWAY | -0.075 | 1.85E-03 | 7.90E-03 |
| KEGG_HYPERTROPHIC_CARDIOMYOPATHY_HCM | -0.059 | 1.78E-03 | 7.90E-03 |
| KEGG_STARCH_AND_SUCROSE_METABOLISM | 0.076 | 1.91E-03 | 8.00E-03 |
| KEGG_ASCORBATE_AND_ALDARATE_METABOLISM | 0.196 | 1.98E-03 | 8.08E-03 |
| KEGG_TYROSINE_METABOLISM | 0.292 | 2.17E-03 | 8.50E-03 |
| KEGG_FC_GAMMA_R_MEDIATED_PHAGOCYTOSIS | -0.024 | 2.13E-03 | 8.50E-03 |
| KEGG_FRUCTOSE_AND_MANNOSE_METABOLISM | 0.177 | 2.55E-03 | 9.40E-03 |
| KEGG_APOPTOSIS | -0.024 | 2.45E-03 | 9.40E-03 |
| KEGG_NEUROTROPHIN_SIGNALING_PATHWAY | -0.047 | 2.56E-03 | 9.40E-03 |
| KEGG_PYRIMIDINE_METABOLISM | -0.023 | 2.74E-03 | 9.89E-03 |
| KEGG_VEGF_SIGNALING_PATHWAY | -0.081 | 3.01E-03 | 1.07E-02 |
| KEGG_CITRATE_CYCLE_TCA_CYCLE | -0.034 | 3.15E-03 | 1.09E-02 |
| KEGG_PENTOSE_AND_GLUCURONATE_INTERCONVERSIONS | 0.175 | 3.64E-03 | 1.24E-02 |
| KEGG_PORPHYRIN_AND_CHLOROPHYLL_METABOLISM | 0.128 | 3.74E-03 | 1.25E-02 |
| KEGG_ADHERENS_JUNCTION | 0.016 | 4.07E-03 | 1.34E-02 |
| KEGG_B_CELL_RECEPTOR_SIGNALING_PATHWAY | -0.079 | 4.16E-03 | 1.34E-02 |
| KEGG_GAP_JUNCTION | 0.032 | 4.28E-03 | 1.36E-02 |
| KEGG_HISTIDINE_METABOLISM | 0.185 | 4.73E-03 | 1.48E-02 |
| KEGG_INOSITOL_PHOSPHATE_METABOLISM | 0.120 | 4.83E-03 | 1.48E-02 |
| KEGG_COLORECTAL_CANCER | -0.146 | 4.98E-03 | 1.50E-02 |
| KEGG_ERBB_SIGNALING_PATHWAY | -0.063 | 5.09E-03 | 1.51E-02 |
| KEGG_LONG_TERM_DEPRESSION | -0.101 | 6.00E-03 | 1.75E-02 |
| KEGG_LINOLEIC_ACID_METABOLISM | 0.253 | 6.54E-03 | 1.88E-02 |
| KEGG_LONG_TERM_POTENTIATION | 0.022 | 6.68E-03 | 1.89E-02 |
| KEGG_ALANINE_ASPARTATE_AND_GLUTAMATE_METABOLISM | 0.092 | 7.19E-03 | 1.93E-02 |
| KEGG_CELL_ADHESION_MOLECULES_CAMS | 0.020 | 6.93E-03 | 1.93E-02 |
| KEGG_GNRH_SIGNALING_PATHWAY | 0.008 | 7.09E-03 | 1.93E-02 |
| KEGG_CHRONIC_MYELOID_LEUKEMIA | -0.058 | 7.22E-03 | 1.93E-02 |
| KEGG_PROPANOATE_METABOLISM | 0.084 | 7.53E-03 | 1.98E-02 |
| KEGG_NON_SMALL_CELL_LUNG_CANCER | -0.023 | 7.67E-03 | 1.99E-02 |
| KEGG_PHOSPHATIDYLINOSITOL_SIGNALING_SYSTEM | 0.122 | 7.86E-03 | 2.01E-02 |
| KEGG_ARACHIDONIC_ACID_METABOLISM | 0.157 | 8.09E-03 | 2.01E-02 |
| KEGG_T_CELL_RECEPTOR_SIGNALING_PATHWAY | -0.073 | 8.03E-03 | 2.01E-02 |
| KEGG_RENAL_CELL_CARCINOMA | -0.032 | 8.63E-03 | 2.12E-02 |
| KEGG_TRYPTOPHAN_METABOLISM | 0.158 | 8.89E-03 | 2.15E-02 |
| KEGG_TOLL_LIKE_RECEPTOR_SIGNALING_PATHWAY | -0.127 | 8.98E-03 | 2.15E-02 |
| KEGG_ABC_TRANSPORTERS | -0.052 | 9.16E-03 | 2.16E-02 |
| KEGG_PANCREATIC_CANCER | -0.029 | 1.06E-02 | 2.47E-02 |
| KEGG_PATHOGENIC_ESCHERICHIA_COLI_INFECTION | 0.017 | 1.18E-02 | 2.72E-02 |
| KEGG_NATURAL_KILLER_CELL_MEDIATED_CYTOTOXICITY | -0.031 | 1.20E-02 | 2.72E-02 |
| KEGG_P53_SIGNALING_PATHWAY | -0.046 | 1.24E-02 | 2.75E-02 |
| KEGG_ADIPOCYTOKINE_SIGNALING_PATHWAY | 0.108 | 1.24E-02 | 2.75E-02 |
| KEGG_OOCYTE_MEIOSIS | -0.028 | 1.28E-02 | 2.78E-02 |
| KEGG_VASOPRESSIN_REGULATED_WATER_REABSORPTION | -0.054 | 1.30E-02 | 2.78E-02 |
| KEGG_VIRAL_MYOCARDITIS | -0.062 | 1.30E-02 | 2.78E-02 |
| KEGG_RNA_DEGRADATION | -0.122 | 1.33E-02 | 2.79E-02 |
| KEGG_LEISHMANIA_INFECTION | -0.071 | 1.33E-02 | 2.79E-02 |
| KEGG_N_GLYCAN_BIOSYNTHESIS | -0.206 | 1.37E-02 | 2.83E-02 |
| KEGG_DILATED_CARDIOMYOPATHY | -0.061 | 1.46E-02 | 2.97E-02 |
| KEGG_EPITHELIAL_CELL_SIGNALING_IN_HELICOBACTER_PYLORI_INFECTION | -0.064 | 1.47E-02 | 2.98E-02 |
| KEGG_PROGESTERONE_MEDIATED_OOCYTE_MATURATION | -0.109 | 1.64E-02 | 3.29E-02 |
| KEGG_GLYCEROPHOSPHOLIPID_METABOLISM | 0.090 | 1.80E-02 | 3.57E-02 |
| KEGG_FC_EPSILON_RI_SIGNALING_PATHWAY | -0.067 | 1.87E-02 | 3.63E-02 |
| KEGG_MELANOGENESIS | -0.021 | 1.87E-02 | 3.63E-02 |
| KEGG_GALACTOSE_METABOLISM | 0.010 | 2.03E-02 | 3.81E-02 |
| KEGG_GLYCEROLIPID_METABOLISM | 0.099 | 2.07E-02 | 3.81E-02 |
| KEGG_NICOTINATE_AND_NICOTINAMIDE_METABOLISM | 0.331 | 2.02E-02 | 3.81E-02 |
| KEGG_PROTEIN_EXPORT | -0.060 | 2.04E-02 | 3.81E-02 |
| KEGG_MTOR_SIGNALING_PATHWAY | -0.070 | 2.07E-02 | 3.81E-02 |
| KEGG_PRIMARY_BILE_ACID_BIOSYNTHESIS | 0.237 | 2.10E-02 | 3.82E-02 |
| KEGG_NITROGEN_METABOLISM | 0.178 | 2.20E-02 | 3.96E-02 |
| KEGG_PROSTATE_CANCER | -0.023 | 2.30E-02 | 4.11E-02 |
| KEGG_PENTOSE_PHOSPHATE_PATHWAY | 0.132 | 2.65E-02 | 4.69E-02 |
| KEGG_AMINOACYL_TRNA_BIOSYNTHESIS | -0.071 | 2.73E-02 | 4.78E-02 |
| KEGG_PROTEASOME | -0.092 | 2.81E-02 | 4.84E-02 |
| KEGG_CELL_CYCLE | 0.013 | 2.81E-02 | 4.84E-02 |
| KEGG_VIBRIO_CHOLERAE_INFECTION | 0.012 | 3.06E-02 | 5.20E-02 |
| KEGG_GLIOMA | -0.012 | 3.08E-02 | 5.20E-02 |
| KEGG_ALDOSTERONE_REGULATED_SODIUM_REABSORPTION | -0.002 | 3.16E-02 | 5.28E-02 |
| KEGG_BETA_ALANINE_METABOLISM | 0.203 | 3.19E-02 | 5.29E-02 |
| KEGG_LYSINE_DEGRADATION | 0.068 | 3.35E-02 | 5.49E-02 |
| KEGG_GLYOXYLATE_AND_DICARBOXYLATE_METABOLISM | 0.011 | 3.37E-02 | 5.49E-02 |
| KEGG_CYTOKINE_CYTOKINE_RECEPTOR_INTERACTION | 0.036 | 3.50E-02 | 5.64E-02 |
| KEGG_SYSTEMIC_LUPUS_ERYTHEMATOSUS | -0.015 | 3.53E-02 | 5.66E-02 |
| KEGG_SNARE_INTERACTIONS_IN_VESICULAR_TRANSPORT | -0.100 | 3.79E-02 | 6.01E-02 |
| KEGG_STEROID_BIOSYNTHESIS | 0.089 | 4.21E-02 | 6.62E-02 |
| KEGG_PRION_DISEASES | 0.041 | 4.56E-02 | 7.11E-02 |
| KEGG_BASE_EXCISION_REPAIR | -0.057 | 4.80E-02 | 7.30E-02 |
| KEGG_TYPE_II_DIABETES_MELLITUS | -0.062 | 4.78E-02 | 7.30E-02 |
| KEGG_ACUTE_MYELOID_LEUKEMIA | -0.043 | 4.77E-02 | 7.30E-02 |
| KEGG_PHENYLALANINE_METABOLISM | 0.178 | 4.89E-02 | 7.38E-02 |
| KEGG_AMYOTROPHIC_LATERAL_SCLEROSIS_ALS | -0.060 | 5.20E-02 | 7.78E-02 |
| KEGG_ENDOMETRIAL_CANCER | -0.098 | 5.24E-02 | 7.78E-02 |
| KEGG_OTHER_GLYCAN_DEGRADATION | -0.139 | 5.70E-02 | 8.39E-02 |
| KEGG_ANTIGEN_PROCESSING_AND_PRESENTATION | -0.094 | 5.74E-02 | 8.39E-02 |
| KEGG_JAK_STAT_SIGNALING_PATHWAY | 0.017 | 5.94E-02 | 8.61E-02 |
| KEGG_CARDIAC_MUSCLE_CONTRACTION | 0.008 | 6.14E-02 | 8.71E-02 |
| KEGG_TGF_BETA_SIGNALING_PATHWAY | -0.071 | 6.16E-02 | 8.71E-02 |
| KEGG_HEMATOPOIETIC_CELL_LINEAGE | 0.035 | 6.14E-02 | 8.71E-02 |
| KEGG_NOD_LIKE_RECEPTOR_SIGNALING_PATHWAY | 0.004 | 6.39E-02 | 8.98E-02 |
| KEGG_CYTOSOLIC_DNA_SENSING_PATHWAY | 0.039 | 6.92E-02 | 9.64E-02 |
| KEGG_DNA_REPLICATION | -0.015 | 7.24E-02 | 9.94E-02 |
| KEGG_MISMATCH_REPAIR | 0.001 | 7.24E-02 | 9.94E-02 |
| KEGG_NUCLEOTIDE_EXCISION_REPAIR | 0.028 | 7.73E-02 | 1.05E-01 |
| KEGG_LIMONENE_AND_PINENE_DEGRADATION | 0.263 | 7.89E-02 | 1.06E-01 |
| KEGG_MATURITY_ONSET_DIABETES_OF_THE_YOUNG | 0.316 | 7.95E-02 | 1.06E-01 |
| KEGG_MELANOMA | -0.018 | 7.97E-02 | 1.06E-01 |
| KEGG_RIG_I_LIKE_RECEPTOR_SIGNALING_PATHWAY | 0.016 | 8.11E-02 | 1.07E-01 |
| KEGG_NEUROACTIVE_LIGAND_RECEPTOR_INTERACTION | -0.145 | 1.04E-01 | 1.37E-01 |
| KEGG_SPHINGOLIPID_METABOLISM | 0.014 | 1.14E-01 | 1.49E-01 |
| KEGG_BIOSYNTHESIS_OF_UNSATURATED_FATTY_ACIDS | 0.121 | 1.21E-01 | 1.57E-01 |
| KEGG_CYSTEINE_AND_METHIONINE_METABOLISM | 0.061 | 1.29E-01 | 1.65E-01 |
| KEGG_SELENOAMINO_ACID_METABOLISM | 0.041 | 1.30E-01 | 1.65E-01 |
| KEGG_THYROID_CANCER | -0.108 | 1.30E-01 | 1.65E-01 |
| KEGG_PRIMARY_IMMUNODEFICIENCY | 0.159 | 1.37E-01 | 1.72E-01 |
| KEGG_RIBOFLAVIN_METABOLISM | 0.134 | 1.49E-01 | 1.86E-01 |
| KEGG_BLADDER_CANCER | 0.003 | 1.66E-01 | 2.06E-01 |
| KEGG_GLYCOSAMINOGLYCAN_DEGRADATION | 0.040 | 1.79E-01 | 2.21E-01 |
| KEGG_ETHER_LIPID_METABOLISM | 0.012 | 1.85E-01 | 2.27E-01 |
| KEGG_BASAL_CELL_CARCINOMA | -0.512 | 1.97E-01 | 2.40E-01 |
| KEGG_GLYCOSPHINGOLIPID_BIOSYNTHESIS_GLOBO_SERIES | -0.232 | 2.16E-01 | 2.56E-01 |
| KEGG_SULFUR_METABOLISM | 0.039 | 2.15E-01 | 2.56E-01 |
| KEGG_NOTCH_SIGNALING_PATHWAY | 0.020 | 2.12E-01 | 2.56E-01 |
| KEGG_RENIN_ANGIOTENSIN_SYSTEM | 0.094 | 2.15E-01 | 2.56E-01 |
| KEGG_TERPENOID_BACKBONE_BIOSYNTHESIS | 0.176 | 2.41E-01 | 2.85E-01 |
| KEGG_ONE_CARBON_POOL_BY_FOLATE | 0.051 | 2.45E-01 | 2.87E-01 |
| KEGG_OLFACTORY_TRANSDUCTION | 0.158 | 2.48E-01 | 2.89E-01 |
| KEGG_HOMOLOGOUS_RECOMBINATION | -0.092 | 2.54E-01 | 2.92E-01 |
| KEGG_PROXIMAL_TUBULE_BICARBONATE_RECLAMATION | 0.065 | 2.52E-01 | 2.92E-01 |
| KEGG_FOLATE_BIOSYNTHESIS | 0.185 | 2.57E-01 | 2.94E-01 |
| KEGG_NON_HOMOLOGOUS_END_JOINING | -0.295 | 2.82E-01 | 3.18E-01 |
| KEGG_REGULATION_OF_AUTOPHAGY | -0.035 | 2.81E-01 | 3.18E-01 |
| KEGG_VALINE_LEUCINE_AND_ISOLEUCINE_BIOSYNTHESIS | -0.006 | 3.58E-01 | 4.01E-01 |
| KEGG_PANTOTHENATE_AND_COA_BIOSYNTHESIS | -0.053 | 3.66E-01 | 4.08E-01 |
| KEGG_ASTHMA | 0.099 | 3.91E-01 | 4.33E-01 |
| KEGG_DORSO_VENTRAL_AXIS_FORMATION | -0.096 | 4.04E-01 | 4.45E-01 |
| KEGG_HEDGEHOG_SIGNALING_PATHWAY | 0.151 | 4.31E-01 | 4.72E-01 |
| KEGG_RNA_POLYMERASE | -0.100 | 4.55E-01 | 4.95E-01 |
| KEGG_TASTE_TRANSDUCTION | 0.080 | 4.59E-01 | 4.97E-01 |
| KEGG_O_GLYCAN_BIOSYNTHESIS | -0.198 | 4.79E-01 | 5.15E-01 |
| KEGG_INTESTINAL_IMMUNE_NETWORK_FOR_IGA_PRODUCTION | -0.190 | 5.03E-01 | 5.32E-01 |
| KEGG_AUTOIMMUNE_THYROID_DISEASE | -0.015 | 5.03E-01 | 5.32E-01 |
| KEGG_ALLOGRAFT_REJECTION | -0.015 | 5.03E-01 | 5.32E-01 |
| KEGG_TYPE_I_DIABETES_MELLITUS | 0.217 | 5.49E-01 | 5.77E-01 |
| KEGG_BASAL_TRANSCRIPTION_FACTORS | -0.146 | 5.63E-01 | 5.88E-01 |
| KEGG_GLYCOSYLPHOSPHATIDYLINOSITOL_GPI_ANCHOR_BIOSYNTHESIS | 0.011 | 5.88E-01 | 6.11E-01 |
| KEGG_ALPHA_LINOLENIC_ACID_METABOLISM | 0.197 | 6.32E-01 | 6.53E-01 |
| KEGG_GLYCOSPHINGOLIPID_BIOSYNTHESIS_GANGLIO_SERIES | 0.067 | 6.99E-01 | 7.19E-01 |
| KEGG_TAURINE_AND_HYPOTAURINE_METABOLISM | 0.089 | 7.35E-01 | 7.52E-01 |
| KEGG_GLYCOSAMINOGLYCAN_BIOSYNTHESIS_CHONDROITIN_SULFATE | -0.244 | 8.07E-01 | 8.21E-01 |
| KEGG_CIRCADIAN_RHYTHM_MAMMAL | 0.153 | 8.79E-01 | 8.88E-01 |
| KEGG_GLYCOSPHINGOLIPID_BIOSYNTHESIS_LACTO_AND_NEOLACTO_SERIES | -0.074 | 9.41E-01 | 9.46E-01 |
| KEGG_GRAFT_VERSUS_HOST_DISEASE | -0.031 | 9.75E-01 | 9.75E-01 |

^1^Averaged Spearman's rank correlation coefficients of all the mRNA-protein pairs in the corresponding pathway.

^2^Kolmogorov-Smirnov test *P* value.

^3^Benjamini-Hochberg adjusted *P* value from Kolmogorov-Smirnov test.

| Table S3. KEGG annotation of differentially  expressed proteins among the 3 subgroups | | |
| --- | --- | --- |
| KEGG annotation | **Proteins involved^1^** | **Subgroup^2^** |
| KEGG_ACUTE_MYELOID_LEUKEMIA | PIK3CD | S1 |
| KEGG_ADHERENS_JUNCTION | ERBB2 | S1 |
| KEGG_ADIPOCYTOKINE_SIGNALING_PATHWAY | NPY | S1 |
| KEGG_ALDOSTERONE_REGULATED_SODIUM_REABSORPTION | PIK3CD | S1 |
| KEGG_ALLOGRAFT_REJECTION | HLA-A;HLA-C;HLA-DQA1;HLA-DQB1 | S1 |
| KEGG_AMINO_SUGAR_AND_NUCLEOTIDE_SUGAR_METABOLISM | UGP2 | S1 |
| KEGG_ANTIGEN_PROCESSING_AND_PRESENTATION | HLA-A;HLA-C;HLA-DQA1;HLA-DQB1 | S1 |
| KEGG_APOPTOSIS | PIK3CD;XIAP | S1 |
| KEGG_ASTHMA | HLA-DQA1;HLA-DQB1 | S1 |
| KEGG_AUTOIMMUNE_THYROID_DISEASE | HLA-A;HLA-C;HLA-DQA1;HLA-DQB1 | S1 |
| KEGG_B_CELL_RECEPTOR_SIGNALING_PATHWAY | PIK3CD | S1 |
| KEGG_BASAL_TRANSCRIPTION_FACTORS | GTF2F2 | S1 |
| KEGG_BLADDER_CANCER | ERBB2 | S1 |
| KEGG_CALCIUM_SIGNALING_PATHWAY | ERBB2;GNA14 | S1 |
| KEGG_CARDIAC_MUSCLE_CONTRACTION | TPM1 | S1 |
| KEGG_CELL_ADHESION_MOLECULES_CAMS | HLA-A;HLA-C;HLA-DQA1;HLA-DQB1;NCAM1 | S1 |
| KEGG_CHEMOKINE_SIGNALING_PATHWAY | CCL19;CCL21;PIK3CD | S1 |
| KEGG_CHRONIC_MYELOID_LEUKEMIA | PIK3CD | S1 |
| KEGG_COLORECTAL_CANCER | PIK3CD | S1 |
| KEGG_COMPLEMENT_AND_COAGULATION_CASCADES | F13B | S1 |
| KEGG_CYTOKINE_CYTOKINE_RECEPTOR_INTERACTION | CCL19;CCL21 | S1 |
| KEGG_DILATED_CARDIOMYOPATHY | TPM1 | S1 |
| KEGG_DNA_REPLICATION | RFC1 | S1 |
| KEGG_ECM_RECEPTOR_INTERACTION | GP1BB;LAMB3 | S1 |
| KEGG_ENDOCYTOSIS | ACAP2;CHMP2B;HLA-A;HLA-C | S1 |
| KEGG_ENDOMETRIAL_CANCER | ERBB2;PIK3CD | S1 |
| KEGG_ERBB_SIGNALING_PATHWAY | ERBB2;PIK3CD | S1 |
| KEGG_FC_EPSILON_RI_SIGNALING_PATHWAY | PIK3CD | S1 |
| KEGG_FC_GAMMA_R_MEDIATED_PHAGOCYTOSIS | PIK3CD | S1 |
| KEGG_FOCAL_ADHESION | ERBB2;LAMB3;PIK3CD;XIAP | S1 |
| KEGG_GALACTOSE_METABOLISM | UGP2 | S1 |
| KEGG_GAP_JUNCTION | TUBAL3 | S1 |
| KEGG_GLIOMA | PIK3CD | S1 |
| KEGG_GLYCOLYSIS_GLUCONEOGENESIS | PGAM2 | S1 |
| KEGG_GLYCOSYLPHOSPHATIDYLINOSITOL_GPI_ANCHOR_BIOSYNTHESIS | PIGK | S1 |
| KEGG_GRAFT_VERSUS_HOST_DISEASE | HLA-A;HLA-C;HLA-DQA1;HLA-DQB1 | S1 |
| KEGG_HEMATOPOIETIC_CELL_LINEAGE | GP1BB | S1 |
| KEGG_HYPERTROPHIC_CARDIOMYOPATHY_HCM | TPM1 | S1 |
| KEGG_INOSITOL_PHOSPHATE_METABOLISM | INPP4A;PIK3CD | S1 |
| KEGG_INSULIN_SIGNALING_PATHWAY | PIK3CD | S1 |
| KEGG_INTESTINAL_IMMUNE_NETWORK_FOR_IGA_PRODUCTION | HLA-DQA1;HLA-DQB1 | S1 |
| KEGG_JAK_STAT_SIGNALING_PATHWAY | IRF9;PIK3CD | S1 |
| KEGG_LEISHMANIA_INFECTION | HLA-DQA1;HLA-DQB1;NCF2 | S1 |
| KEGG_LEUKOCYTE_TRANSENDOTHELIAL_MIGRATION | NCF2;PIK3CD | S1 |
| KEGG_LYSOSOME | CLN5 | S1 |
| KEGG_MAPK_SIGNALING_PATHWAY | FGF2 | S1 |
| KEGG_MELANOMA | FGF2;PIK3CD | S1 |
| KEGG_MISMATCH_REPAIR | RFC1 | S1 |
| KEGG_MTOR_SIGNALING_PATHWAY | PIK3CD;RICTOR | S1 |
| KEGG_NATURAL_KILLER_CELL_MEDIATED_CYTOTOXICITY | HLA-A;HLA-C;PIK3CD;SH2D1A;ZAP70 | S1 |
| KEGG_NEUROACTIVE_LIGAND_RECEPTOR_INTERACTION | TSPO | S1 |
| KEGG_NEUROTROPHIN_SIGNALING_PATHWAY | PIK3CD | S1 |
| KEGG_NICOTINATE_AND_NICOTINAMIDE_METABOLISM | NNT | S1 |
| KEGG_NITROGEN_METABOLISM | CA14 | S1 |
| KEGG_NOD_LIKE_RECEPTOR_SIGNALING_PATHWAY | PSTPIP1;XIAP | S1 |
| KEGG_NON_SMALL_CELL_LUNG_CANCER | ERBB2;PIK3CD | S1 |
| KEGG_NUCLEOTIDE_EXCISION_REPAIR | CETN2;DDB2;RFC1 | S1 |
| KEGG_OXIDATIVE_PHOSPHORYLATION | COX15 | S1 |
| KEGG_P53_SIGNALING_PATHWAY | DDB2 | S1 |
| KEGG_PANCREATIC_CANCER | ERBB2;PIK3CD | S1 |
| KEGG_PARKINSONS_DISEASE | UBE2L3 | S1 |
| KEGG_PATHOGENIC_ESCHERICHIA_COLI_INFECTION | TUBAL3 | S1 |
| KEGG_PATHWAYS_IN_CANCER | ERBB2;FGF2;LAMB3;PIK3CD;TRAF4;XIAP | S1 |
| KEGG_PENTOSE_AND_GLUCURONATE_INTERCONVERSIONS | UGP2 | S1 |
| KEGG_PEROXISOME | MPV17 | S1 |
| KEGG_PHENYLALANINE_METABOLISM | HPD | S1 |
| KEGG_PHOSPHATIDYLINOSITOL_SIGNALING_SYSTEM | INPP4A;PIK3CD | S1 |
| KEGG_PORPHYRIN_AND_CHLOROPHYLL_METABOLISM | COX15 | S1 |
| KEGG_PRIMARY_IMMUNODEFICIENCY | ZAP70 | S1 |
| KEGG_PRION_DISEASES | NCAM1 | S1 |
| KEGG_PROGESTERONE_MEDIATED_OOCYTE_MATURATION | PIK3CD | S1 |
| KEGG_PROSTATE_CANCER | ERBB2;PIK3CD | S1 |
| KEGG_PURINE_METABOLISM | ENTPD1;PDE10A | S1 |
| KEGG_PYRIMIDINE_METABOLISM | ENTPD1;TXNRD2 | S1 |
| KEGG_REGULATION_OF_ACTIN_CYTOSKELETON | FGF2;PIK3CD | S1 |
| KEGG_RENAL_CELL_CARCINOMA | PIK3CD | S1 |
| KEGG_RIBOSOME | RPL29 | S1 |
| KEGG_SMALL_CELL_LUNG_CANCER | LAMB3;PIK3CD;TRAF4;XIAP | S1 |
| KEGG_SNARE_INTERACTIONS_IN_VESICULAR_TRANSPORT | VAMP3 | S1 |
| KEGG_STARCH_AND_SUCROSE_METABOLISM | UGP2 | S1 |
| KEGG_SYSTEMIC_LUPUS_ERYTHEMATOSUS | HLA-DQA1;HLA-DQB1 | S1 |
| KEGG_T_CELL_RECEPTOR_SIGNALING_PATHWAY | GRAP2;PIK3CD;ZAP70 | S1 |
| KEGG_TIGHT_JUNCTION | EPB41L1 | S1 |
| KEGG_TOLL_LIKE_RECEPTOR_SIGNALING_PATHWAY | PIK3CD | S1 |
| KEGG_TYPE_I_DIABETES_MELLITUS | HLA-A;HLA-C;HLA-DQA1;HLA-DQB1 | S1 |
| KEGG_TYPE_II_DIABETES_MELLITUS | PIK3CD | S1 |
| KEGG_TYROSINE_METABOLISM | HPD | S1 |
| KEGG_UBIQUITIN_MEDIATED_PROTEOLYSIS | DDB2;UBE2L3;XIAP | S1 |
| KEGG_VEGF_SIGNALING_PATHWAY | PIK3CD | S1 |
| KEGG_VIRAL_MYOCARDITIS | HLA-A;HLA-C;HLA-DQA1;HLA-DQB1 | S1 |
| KEGG_ACUTE_MYELOID_LEUKEMIA | CEBPA;STAT3 | S2 |
| KEGG_ADHERENS_JUNCTION | AFDN;CTNNA2 | S2 |
| KEGG_ADIPOCYTOKINE_SIGNALING_PATHWAY | ACSL1;NFKBIE;STAT3 | S2 |
| KEGG_ALANINE_ASPARTATE_AND_GLUTAMATE_METABOLISM | ASPA | S2 |
| KEGG_ALDOSTERONE_REGULATED_SODIUM_REABSORPTION | PDPK1 | S2 |
| KEGG_ALLOGRAFT_REJECTION | HLA-A;HLA-B;HLA-DMB;HLA-F | S2 |
| KEGG_ALPHA_LINOLENIC_ACID_METABOLISM | ACOX1 | S2 |
| KEGG_ALZHEIMERS_DISEASE | ITPR1;UQCR11 | S2 |
| KEGG_AMINOACYL_TRNA_BIOSYNTHESIS | MTFMT | S2 |
| KEGG_ANTIGEN_PROCESSING_AND_PRESENTATION | HLA-A;HLA-B;HLA-DMB;HLA-F | S2 |
| KEGG_APOPTOSIS | IL1R1 | S2 |
| KEGG_ARRHYTHMOGENIC_RIGHT_VENTRICULAR_CARDIOMYOPATHY_ARVC | CTNNA2 | S2 |
| KEGG_ASCORBATE_AND_ALDARATE_METABOLISM | UGT1A1 | S2 |
| KEGG_ASTHMA | FCER1G;HLA-DMB | S2 |
| KEGG_AUTOIMMUNE_THYROID_DISEASE | HLA-A;HLA-B;HLA-DMB;HLA-F | S2 |
| KEGG_B_CELL_RECEPTOR_SIGNALING_PATHWAY | NFKBIE | S2 |
| KEGG_BASAL_TRANSCRIPTION_FACTORS | GTF2A2 | S2 |
| KEGG_BASE_EXCISION_REPAIR | SMUG1 | S2 |
| KEGG_BETA_ALANINE_METABOLISM | ECHS1 | S2 |
| KEGG_BIOSYNTHESIS_OF_UNSATURATED_FATTY_ACIDS | ACOX1 | S2 |
| KEGG_BUTANOATE_METABOLISM | ECHS1 | S2 |
| KEGG_CALCIUM_SIGNALING_PATHWAY | ITPR1;SPHK2 | S2 |
| KEGG_CARDIAC_MUSCLE_CONTRACTION | UQCR11 | S2 |
| KEGG_CELL_ADHESION_MOLECULES_CAMS | HLA-A;HLA-B;HLA-DMB;HLA-F | S2 |
| KEGG_CELL_CYCLE | ANAPC7;MCM4;RBX1 | S2 |
| KEGG_CHEMOKINE_SIGNALING_PATHWAY | PREX1;STAT3 | S2 |
| KEGG_COMPLEMENT_AND_COAGULATION_CASCADES | VWF | S2 |
| KEGG_CYTOKINE_CYTOKINE_RECEPTOR_INTERACTION | IL1R1 | S2 |
| KEGG_DNA_REPLICATION | MCM4 | S2 |
| KEGG_DRUG_METABOLISM_CYTOCHROME_P450 | AOX1;GSTT2;UGT1A1 | S2 |
| KEGG_DRUG_METABOLISM_OTHER_ENZYMES | NAT1;UGT1A1;UPP1 | S2 |
| KEGG_ECM_RECEPTOR_INTERACTION | TNXB;VWF | S2 |
| KEGG_ENDOCYTOSIS | AP2A2;HLA-A;HLA-B;HLA-F | S2 |
| KEGG_ENDOMETRIAL_CANCER | CTNNA2;PDPK1 | S2 |
| KEGG_FATTY_ACID_METABOLISM | ACOX1;ACSL1;ECHS1 | S2 |
| KEGG_FC_EPSILON_RI_SIGNALING_PATHWAY | FCER1G;PDPK1 | S2 |
| KEGG_FC_GAMMA_R_MEDIATED_PHAGOCYTOSIS | SPHK2 | S2 |
| KEGG_FOCAL_ADHESION | PDPK1;TNXB;VWF | S2 |
| KEGG_FRUCTOSE_AND_MANNOSE_METABOLISM | MTMR6 | S2 |
| KEGG_GAP_JUNCTION | ITPR1 | S2 |
| KEGG_GLUTATHIONE_METABOLISM | GSTT2 | S2 |
| KEGG_GLYCOLYSIS_GLUCONEOGENESIS | ENO3 | S2 |
| KEGG_GLYCOSYLPHOSPHATIDYLINOSITOL_GPI_ANCHOR_BIOSYNTHESIS | PIGO | S2 |
| KEGG_GNRH_SIGNALING_PATHWAY | ITPR1 | S2 |
| KEGG_GRAFT_VERSUS_HOST_DISEASE | HLA-A;HLA-B;HLA-DMB;HLA-F | S2 |
| KEGG_HEMATOPOIETIC_CELL_LINEAGE | IL1R1 | S2 |
| KEGG_HISTIDINE_METABOLISM | ASPA | S2 |
| KEGG_HUNTINGTONS_DISEASE | AP2A2;ITPR1;POLR2L;UQCR11 | S2 |
| KEGG_INSULIN_SIGNALING_PATHWAY | PDPK1 | S2 |
| KEGG_INTESTINAL_IMMUNE_NETWORK_FOR_IGA_PRODUCTION | HLA-DMB | S2 |
| KEGG_JAK_STAT_SIGNALING_PATHWAY | STAT3 | S2 |
| KEGG_LEISHMANIA_INFECTION | HLA-DMB | S2 |
| KEGG_LEUKOCYTE_TRANSENDOTHELIAL_MIGRATION | AFDN;CTNNA2 | S2 |
| KEGG_LIMONENE_AND_PINENE_DEGRADATION | ECHS1 | S2 |
| KEGG_LONG_TERM_DEPRESSION | ITPR1 | S2 |
| KEGG_LONG_TERM_POTENTIATION | ITPR1;PPP1R1A | S2 |
| KEGG_LYSINE_DEGRADATION | ECHS1 | S2 |
| KEGG_LYSOSOME | NPC1 | S2 |
| KEGG_MAPK_SIGNALING_PATHWAY | IL1R1 | S2 |
| KEGG_METABOLISM_OF_XENOBIOTICS_BY_CYTOCHROME_P450 | GSTT2;UGT1A1 | S2 |
| KEGG_MTOR_SIGNALING_PATHWAY | MLST8;PDPK1 | S2 |
| KEGG_N_GLYCAN_BIOSYNTHESIS | ALG14 | S2 |
| KEGG_NATURAL_KILLER_CELL_MEDIATED_CYTOTOXICITY | FCER1G;HLA-A;HLA-B | S2 |
| KEGG_NEUROACTIVE_LIGAND_RECEPTOR_INTERACTION | GZMA | S2 |
| KEGG_NEUROTROPHIN_SIGNALING_PATHWAY | NFKBIE;PDPK1 | S2 |
| KEGG_NICOTINATE_AND_NICOTINAMIDE_METABOLISM | AOX1 | S2 |
| KEGG_NITROGEN_METABOLISM | CA3 | S2 |
| KEGG_NON_SMALL_CELL_LUNG_CANCER | PDPK1 | S2 |
| KEGG_NUCLEOTIDE_EXCISION_REPAIR | RBX1;XPC | S2 |
| KEGG_ONE_CARBON_POOL_BY_FOLATE | MTFMT;MTHFR | S2 |
| KEGG_OOCYTE_MEIOSIS | ANAPC7;ITPR1;RBX1 | S2 |
| KEGG_OXIDATIVE_PHOSPHORYLATION | UQCR11 | S2 |
| KEGG_P53_SIGNALING_PATHWAY | IGFBP3 | S2 |
| KEGG_PANCREATIC_CANCER | STAT3 | S2 |
| KEGG_PARKINSONS_DISEASE | UQCR11 | S2 |
| KEGG_PATHWAYS_IN_CANCER | CEBPA;CTNNA2;RBX1;STAT3 | S2 |
| KEGG_PENTOSE_AND_GLUCURONATE_INTERCONVERSIONS | UGT1A1 | S2 |
| KEGG_PEROXISOME | ACOX1;ACSL1 | S2 |
| KEGG_PHOSPHATIDYLINOSITOL_SIGNALING_SYSTEM | ITPR1 | S2 |
| KEGG_PORPHYRIN_AND_CHLOROPHYLL_METABOLISM | UGT1A1 | S2 |
| KEGG_PPAR_SIGNALING_PATHWAY | ACOX1;ACSL1;PDPK1 | S2 |
| KEGG_PROGESTERONE_MEDIATED_OOCYTE_MATURATION | ANAPC7 | S2 |
| KEGG_PROPANOATE_METABOLISM | ECHS1 | S2 |
| KEGG_PROSTATE_CANCER | PDPK1 | S2 |
| KEGG_PURINE_METABOLISM | DGUOK;POLR2L | S2 |
| KEGG_PYRIMIDINE_METABOLISM | POLR2L;TXNRD2;UPP1 | S2 |
| KEGG_REGULATION_OF_ACTIN_CYTOSKELETON | ITGAE | S2 |
| KEGG_REGULATION_OF_AUTOPHAGY | GABARAPL1 | S2 |
| KEGG_RENAL_CELL_CARCINOMA | RBX1 | S2 |
| KEGG_RETINOL_METABOLISM | UGT1A1 | S2 |
| KEGG_RIBOFLAVIN_METABOLISM | MTMR6 | S2 |
| KEGG_RIBOSOME | RPL26L1 | S2 |
| KEGG_RNA_DEGRADATION | ENO3;EXOSC8;LSM1 | S2 |
| KEGG_RNA_POLYMERASE | POLR2L | S2 |
| KEGG_SNARE_INTERACTIONS_IN_VESICULAR_TRANSPORT | VAMP5 | S2 |
| KEGG_SPHINGOLIPID_METABOLISM | SPHK2 | S2 |
| KEGG_SPLICEOSOME | CCDC12;CWC15;PRPF38B;SMNDC1 | S2 |
| KEGG_STARCH_AND_SUCROSE_METABOLISM | UGT1A1 | S2 |
| KEGG_STEROID_HORMONE_BIOSYNTHESIS | UGT1A1 | S2 |
| KEGG_SYSTEMIC_LUPUS_ERYTHEMATOSUS | HLA-DMB | S2 |
| KEGG_T_CELL_RECEPTOR_SIGNALING_PATHWAY | NFKBIE;PDPK1 | S2 |
| KEGG_TGF_BETA_SIGNALING_PATHWAY | RBX1;SMAD1 | S2 |
| KEGG_TIGHT_JUNCTION | AFDN;CTNNA2;MYH1 | S2 |
| KEGG_TRYPTOPHAN_METABOLISM | AOX1;ECHS1 | S2 |
| KEGG_TYPE_I_DIABETES_MELLITUS | HLA-A;HLA-B;HLA-DMB;HLA-F | S2 |
| KEGG_TYROSINE_METABOLISM | AOX1;DBH | S2 |
| KEGG_UBIQUITIN_MEDIATED_PROTEOLYSIS | ANAPC7;RBX1;UBE2E1 | S2 |
| KEGG_VALINE_LEUCINE_AND_ISOLEUCINE_DEGRADATION | AOX1;ECHS1 | S2 |
| KEGG_VASCULAR_SMOOTH_MUSCLE_CONTRACTION | ITPR1;MYL6B | S2 |
| KEGG_VEGF_SIGNALING_PATHWAY | SPHK2 | S2 |
| KEGG_VIRAL_MYOCARDITIS | HLA-A;HLA-B;HLA-DMB;HLA-F;MYH1 | S2 |
| KEGG_WNT_SIGNALING_PATHWAY | RBX1 | S2 |
| KEGG_ABC_TRANSPORTERS | ABCC5;ABCC6;ABCD4 | S3 |
| KEGG_ACUTE_MYELOID_LEUKEMIA | RPS6KB2 | S3 |
| KEGG_ADHERENS_JUNCTION | PTPRM | S3 |
| KEGG_ADIPOCYTOKINE_SIGNALING_PATHWAY | NFKBIA | S3 |
| KEGG_ALLOGRAFT_REJECTION | CD40;HLA-DPB1;HLA-DQB1;HLA-DRB1 | S3 |
| KEGG_ALZHEIMERS_DISEASE | GSK3B | S3 |
| KEGG_AMINO_SUGAR_AND_NUCLEOTIDE_SUGAR_METABOLISM | AMDHD2 | S3 |
| KEGG_AMYOTROPHIC_LATERAL_SCLEROSIS_ALS | GPX1 | S3 |
| KEGG_ANTIGEN_PROCESSING_AND_PRESENTATION | HLA-DPB1;HLA-DQB1;HLA-DRB1 | S3 |
| KEGG_APOPTOSIS | NFKBIA;PRKAR2B | S3 |
| KEGG_ARACHIDONIC_ACID_METABOLISM | GGT7;GPX1;LTC4S | S3 |
| KEGG_ARGININE_AND_PROLINE_METABOLISM | GATM | S3 |
| KEGG_ASCORBATE_AND_ALDARATE_METABOLISM | UGT2B7 | S3 |
| KEGG_ASTHMA | CD40;HLA-DPB1;HLA-DQB1;HLA-DRB1 | S3 |
| KEGG_AUTOIMMUNE_THYROID_DISEASE | CD40;HLA-DPB1;HLA-DQB1;HLA-DRB1 | S3 |
| KEGG_AXON_GUIDANCE | CXCL12;GSK3B;NGEF;PLXNB2;RASA1;SEMA4B | S3 |
| KEGG_B_CELL_RECEPTOR_SIGNALING_PATHWAY | GSK3B;NFKBIA;SYK | S3 |
| KEGG_BASAL_CELL_CARCINOMA | GSK3B | S3 |
| KEGG_BASAL_TRANSCRIPTION_FACTORS | TAF10;TAF5 | S3 |
| KEGG_BASE_EXCISION_REPAIR | FEN1;MPG;XRCC1 | S3 |
| KEGG_BIOSYNTHESIS_OF_UNSATURATED_FATTY_ACIDS | ELOVL5;HACD2 | S3 |
| KEGG_BLADDER_CANCER | CDK4 | S3 |
| KEGG_CELL_ADHESION_MOLECULES_CAMS | CD276;CD40;CD58;ESAM;HLA-DPB1;HLA-DQB1;HLA-DRB1;PTPRM | S3 |
| KEGG_CELL_CYCLE | CDC16;CDK4;GSK3B;MCM6;STAG1 | S3 |
| KEGG_CHEMOKINE_SIGNALING_PATHWAY | CXCL12;GNG5;GSK3B;NFKBIA | S3 |
| KEGG_CHRONIC_MYELOID_LEUKEMIA | CDK4;NFKBIA | S3 |
| KEGG_CIRCADIAN_RHYTHM_MAMMAL | CSNK1D | S3 |
| KEGG_COLORECTAL_CANCER | GSK3B | S3 |
| KEGG_COMPLEMENT_AND_COAGULATION_CASCADES | CD55;MASP2 | S3 |
| KEGG_CYTOKINE_CYTOKINE_RECEPTOR_INTERACTION | CD40;CXCL12 | S3 |
| KEGG_CYTOSOLIC_DNA_SENSING_PATHWAY | NFKBIA;POLR1C | S3 |
| KEGG_DNA_REPLICATION | FEN1;MCM6 | S3 |
| KEGG_DRUG_METABOLISM_CYTOCHROME_P450 | ALDH1A3;GSTZ1;UGT2B7 | S3 |
| KEGG_DRUG_METABOLISM_OTHER_ENZYMES | UCK1;UGT2B7 | S3 |
| KEGG_ENDOCYTOSIS | ASAP1;DNM3;USP8;VPS37A | S3 |
| KEGG_ENDOMETRIAL_CANCER | GSK3B | S3 |
| KEGG_EPITHELIAL_CELL_SIGNALING_IN_HELICOBACTER_PYLORI_INFECTION | NFKBIA | S3 |
| KEGG_ERBB_SIGNALING_PATHWAY | GSK3B;RPS6KB2 | S3 |
| KEGG_ETHER_LIPID_METABOLISM | CHPT1 | S3 |
| KEGG_FC_EPSILON_RI_SIGNALING_PATHWAY | SYK | S3 |
| KEGG_FC_GAMMA_R_MEDIATED_PHAGOCYTOSIS | ASAP1;DNM3;RPS6KB2;SYK | S3 |
| KEGG_FOCAL_ADHESION | ARHGAP5;CAV2;GSK3B;PARVG | S3 |
| KEGG_FRUCTOSE_AND_MANNOSE_METABOLISM | PFKP | S3 |
| KEGG_GALACTOSE_METABOLISM | LCT;PFKP | S3 |
| KEGG_GAP_JUNCTION | CSNK1D;GUCY1A2 | S3 |
| KEGG_GLIOMA | CDK4 | S3 |
| KEGG_GLUTATHIONE_METABOLISM | GGT7;GPX1;GSTZ1;RRM2B | S3 |
| KEGG_GLYCEROPHOSPHOLIPID_METABOLISM | CHPT1;PLA2G15;PTDSS2 | S3 |
| KEGG_GLYCINE_SERINE_AND_THREONINE_METABOLISM | GATM | S3 |
| KEGG_GLYCOLYSIS_GLUCONEOGENESIS | ALDH1A3;PFKP | S3 |
| KEGG_GLYCOSAMINOGLYCAN_BIOSYNTHESIS_CHONDROITIN_SULFATE | B3GALT6;B4GALT7;CHST13 | S3 |
| KEGG_GLYCOSAMINOGLYCAN_BIOSYNTHESIS_HEPARAN_SULFATE | B3GALT6;B4GALT7 | S3 |
| KEGG_GLYCOSAMINOGLYCAN_DEGRADATION | SGSH | S3 |
| KEGG_GRAFT_VERSUS_HOST_DISEASE | HLA-DPB1;HLA-DQB1;HLA-DRB1 | S3 |
| KEGG_HEDGEHOG_SIGNALING_PATHWAY | CSNK1D;GSK3B | S3 |
| KEGG_HEMATOPOIETIC_CELL_LINEAGE | CD55;HLA-DRB1 | S3 |
| KEGG_HISTIDINE_METABOLISM | ALDH1A3 | S3 |
| KEGG_HUNTINGTONS_DISEASE | GPX1;SIN3A | S3 |
| KEGG_INOSITOL_PHOSPHATE_METABOLISM | IMPA2 | S3 |
| KEGG_INSULIN_SIGNALING_PATHWAY | EIF4E2;GSK3B;PRKAR2B;RPS6KB2 | S3 |
| KEGG_INTESTINAL_IMMUNE_NETWORK_FOR_IGA_PRODUCTION | CD40;CXCL12;HLA-DPB1;HLA-DQB1;HLA-DRB1 | S3 |
| KEGG_LEISHMANIA_INFECTION | HLA-DPB1;HLA-DQB1;HLA-DRB1;NFKBIA | S3 |
| KEGG_LEUKOCYTE_TRANSENDOTHELIAL_MIGRATION | ARHGAP5;CXCL12;ESAM;NOX3;RASSF5;SIPA1 | S3 |
| KEGG_LONG_TERM_DEPRESSION | GUCY1A2 | S3 |
| KEGG_LYSOSOME | CTSZ;PLA2G15;SGSH | S3 |
| KEGG_MAPK_SIGNALING_PATHWAY | NF1;RASA1 | S3 |
| KEGG_MELANOGENESIS | GSK3B | S3 |
| KEGG_MELANOMA | CDK4 | S3 |
| KEGG_METABOLISM_OF_XENOBIOTICS_BY_CYTOCHROME_P450 | ALDH1A3;GSTZ1;UGT2B7 | S3 |
| KEGG_MTOR_SIGNALING_PATHWAY | EIF4E2;RPS6KB2 | S3 |
| KEGG_N_GLYCAN_BIOSYNTHESIS | ALG8;MAN1B1 | S3 |
| KEGG_NATURAL_KILLER_CELL_MEDIATED_CYTOTOXICITY | LCK;SYK | S3 |
| KEGG_NEUROTROPHIN_SIGNALING_PATHWAY | GSK3B;NFKBIA | S3 |
| KEGG_NOD_LIKE_RECEPTOR_SIGNALING_PATHWAY | NFKBIA | S3 |
| KEGG_NON_HOMOLOGOUS_END_JOINING | FEN1 | S3 |
| KEGG_NON_SMALL_CELL_LUNG_CANCER | CDK4;RASSF5 | S3 |
| KEGG_O_GLYCAN_BIOSYNTHESIS | B4GALT5;GALNT1 | S3 |
| KEGG_OOCYTE_MEIOSIS | CDC16 | S3 |
| KEGG_P53_SIGNALING_PATHWAY | CDK4;RRM2B | S3 |
| KEGG_PANCREATIC_CANCER | CDK4 | S3 |
| KEGG_PARKINSONS_DISEASE | UBE2G1;UBE2G2;UCHL1 | S3 |
| KEGG_PATHWAYS_IN_CANCER | CDK4;GSK3B;NFKBIA;RASSF5 | S3 |
| KEGG_PENTOSE_AND_GLUCURONATE_INTERCONVERSIONS | UGT2B7 | S3 |
| KEGG_PENTOSE_PHOSPHATE_PATHWAY | PFKP | S3 |
| KEGG_PEROXISOME | ABCD4;PEX10;PEX6;PXMP4;SLC25A17 | S3 |
| KEGG_PHENYLALANINE_METABOLISM | ALDH1A3 | S3 |
| KEGG_PHOSPHATIDYLINOSITOL_SIGNALING_SYSTEM | IMPA2 | S3 |
| KEGG_PORPHYRIN_AND_CHLOROPHYLL_METABOLISM | UGT2B7 | S3 |
| KEGG_PPAR_SIGNALING_PATHWAY | APOA5 | S3 |
| KEGG_PRIMARY_IMMUNODEFICIENCY | CD40;LCK | S3 |
| KEGG_PROGESTERONE_MEDIATED_OOCYTE_MATURATION | CDC16 | S3 |
| KEGG_PROSTATE_CANCER | GSK3B;NFKBIA | S3 |
| KEGG_PROXIMAL_TUBULE_BICARBONATE_RECLAMATION | SLC4A4 | S3 |
| KEGG_PURINE_METABOLISM | GDA;GUCY1A2;PDE6D;POLR1C;RRM2B | S3 |
| KEGG_PYRIMIDINE_METABOLISM | CMPK2;POLR1C;RRM2B;UCK1 | S3 |
| KEGG_REGULATION_OF_ACTIN_CYTOSKELETON | PFN2;SSH3 | S3 |
| KEGG_REGULATION_OF_AUTOPHAGY | BECN1 | S3 |
| KEGG_RETINOL_METABOLISM | UGT2B7 | S3 |
| KEGG_RIG_I_LIKE_RECEPTOR_SIGNALING_PATHWAY | NFKBIA | S3 |
| KEGG_RNA_DEGRADATION | EXOSC1;EXOSC4;PAPOLA | S3 |
| KEGG_RNA_POLYMERASE | POLR1C | S3 |
| KEGG_SELENOAMINO_ACID_METABOLISM | GGT7 | S3 |
| KEGG_SMALL_CELL_LUNG_CANCER | CDK4;NFKBIA | S3 |
| KEGG_SNARE_INTERACTIONS_IN_VESICULAR_TRANSPORT | BET1;STX11 | S3 |
| KEGG_SPHINGOLIPID_METABOLISM | SGPP1;SPTLC2 | S3 |
| KEGG_SPLICEOSOME | PQBP1 | S3 |
| KEGG_STARCH_AND_SUCROSE_METABOLISM | UGT2B7 | S3 |
| KEGG_STEROID_HORMONE_BIOSYNTHESIS | STS;UGT2B7 | S3 |
| KEGG_SULFUR_METABOLISM | CHST13 | S3 |
| KEGG_SYSTEMIC_LUPUS_ERYTHEMATOSUS | CD40;HLA-DPB1;HLA-DQB1;HLA-DRB1 | S3 |
| KEGG_T_CELL_RECEPTOR_SIGNALING_PATHWAY | CDK4;GSK3B;LCK;NFKBIA | S3 |
| KEGG_TAURINE_AND_HYPOTAURINE_METABOLISM | GGT7 | S3 |
| KEGG_TERPENOID_BACKBONE_BIOSYNTHESIS | GGPS1 | S3 |
| KEGG_TGF_BETA_SIGNALING_PATHWAY | RPS6KB2 | S3 |
| KEGG_TIGHT_JUNCTION | CDK4 | S3 |
| KEGG_TOLL_LIKE_RECEPTOR_SIGNALING_PATHWAY | CD40;NFKBIA | S3 |
| KEGG_TYPE_I_DIABETES_MELLITUS | HLA-DPB1;HLA-DQB1;HLA-DRB1 | S3 |
| KEGG_TYROSINE_METABOLISM | ALDH1A3;GSTZ1 | S3 |
| KEGG_UBIQUITIN_MEDIATED_PROTEOLYSIS | BIRC6;CDC16;TRIP12;UBE2G1;UBE2G2 | S3 |
| KEGG_VASCULAR_SMOOTH_MUSCLE_CONTRACTION | GUCY1A2 | S3 |
| KEGG_VASOPRESSIN_REGULATED_WATER_REABSORPTION | DYNLL2 | S3 |
| KEGG_VIBRIO_CHOLERAE_INFECTION | KDELR1 | S3 |
| KEGG_VIRAL_MYOCARDITIS | CD40;CD55;HLA-DPB1;HLA-DQB1;HLA-DRB1 | S3 |
| KEGG_WNT_SIGNALING_PATHWAY | GSK3B | S3 |

^1^Differentially expressed proteins in the corresponding pathway.

^2^Subgroups demonstrating the highest expression of the corresponding proteins.

| **Table S4. Top20 proteins in Fig. 2a** | | | | |
| --- | --- | --- | --- | --- |
| **Protein ID** | Gene symbol | *P* | BH adjsuted *P* | KEGG annotation |
| B4DG79 | NA | 8.48E-15 | 1.15E-11 | NA |
| A0A024RAG3 | RALB | 6.25E-14 | 1.70E-11 | Ras signaling pathway;  Rap1 signaling pathway;  Phospholipase D signaling pathway;  Pathways in cancer;  Colorectal cancer;  Pancreatic cancer |
| A5A3E0 | POTEF | 4.47E-14 | 1.70E-11 | NA |
| B4DJ38 | NA | 5.52E-14 | 1.70E-11 | NA |
| I6L8E2 | MCIDAS | 3.34E-14 | 1.70E-11 | NA |
| D6RIE8 | TAF9 | 8.80E-14 | 2.00E-11 | NA |
| B4E218 | NA | 1.33E-13 | 2.41E-11 | NA |
| F4ZW66 | NA | 1.42E-13 | 2.41E-11 | NA |
| Q6GMV3 | PTRHD1 | 2.26E-13 | 3.37E-11 | NA |
| Q8N3F8 | MICALL1 | 2.48E-13 | 3.37E-11 | NA |
| A0A024QZ64 | ALDOC | 4.66E-13 | 4.64E-11 | Glycolysis / Gluconeogenesis;  Pentose phosphate pathway;  Fructose and mannose metabolism;  Metabolic pathways;  Carbon metabolism;  Biosynthesis of amino acids |
| B4DU07 | NA | 4.85E-13 | 4.64E-11 | NA |
| P10114 | RAP2A | 4.33E-13 | 4.64E-11 | Ribosome biogenesis in eukaryotes;  RNA transport |
| Q8N5L8 | RPP25L | 5.12E-13 | 4.64E-11 | NA |
| Q96HA9 | PEX11G | 5.01E-13 | 4.64E-11 | Peroxisome |
| A0A024R223 | VPS13A | 5.81E-13 | 4.95E-11 | NA |
| A0A024RCR2 | GNL1 | 6.22E-13 | 4.98E-11 | NA |
| A0A024R5H6 | PACS1 | 6.96E-13 | 5.07E-11 | NA |
| A6NLH6 | CNIH4 | 7.07E-13 | 5.07E-11 | NA |
| Q8WUK0 | PTPMT1 | 8.90E-13 | 6.06E-11 | NA |

^1^Kruskal-Wallis test *P* value.

^2^Benjamini-Hochberg adjusted *P* value from Kruskal-Wallis test.
